# Unraveling the mechanism of the cadherin-catenin-actin catch bond

**DOI:** 10.1371/journal.pcbi.1006399

**Published:** 2018-08-17

**Authors:** Shishir Adhikari, Jacob Moran, Christopher Weddle, Michael Hinczewski

**Affiliations:** Department of Physics, Case Western Reserve University, Cleveland, Ohio, United States of America; University of Maryland School of Pharmacy, UNITED STATES

## Abstract

The adherens junctions between epithelial cells involve a protein complex formed by E-cadherin, *β*-catenin, *α*-catenin and F-actin. The stability of this complex was a puzzle for many years, since in vitro studies could reconstitute various stable subsets of the individual proteins, but never the entirety. The missing ingredient turned out to be mechanical tension: a recent experiment that applied physiological forces to the complex with an optical tweezer dramatically increased its lifetime, a phenomenon known as catch bonding. However, in the absence of a crystal structure for the full complex, the microscopic details of the catch bond mechanism remain mysterious. Building on structural clues that point to *α*-catenin as the force transducer, we present a quantitative theoretical model for how the catch bond arises, fully accounting for the experimental lifetime distributions. The underlying hypothesis is that force induces a rotational transition between two conformations of *α*-catenin, overcoming a significant energy barrier due to a network of salt bridges. This transition allosterically regulates the energies at the interface between *α*-catenin and F-actin. The model allows us to predict these energetic changes, as well as highlighting the importance of the salt bridge rotational barrier. By stabilizing one of the *α*-catenin states, this barrier could play a role in how the complex responds to additional *in vivo* binding partners like vinculin. Since significant conformational energy barriers are a common feature of other adhesion systems that exhibit catch bonds, our model can be adapted into a general theoretical framework for integrating structure and function in a variety of force-regulated protein complexes.

## Author summary

Epithelial cells that line the surface of cavities in the human body are held together by groups of proteins known as adherens junctions. The cells are always under some level of mechanical tension, and the resulting forces can play a major role in determining junction stability. Our work provides a theoretical model to account for an intriguing recent experiment, where force was found to enhance the lifetime of the bonds between adherens junction proteins. This counter-intuitive strengthening of biological bonds under tension is known as catch bonding. Though observed in a wide variety of proteins responsible for adhesion between cells, the microscopic biophysical origins of this phenomenon are poorly understood. For the case of the adherens junction, our model allows us to use the experimental data to infer a number of previously unknown details, like the energies at the protein interfaces, and the associated protein structural rearrangements. Since the building blocks of the model—bond energy changes induced by protein domain rotations—are common to many adhesion protein systems that exhibit catch bonds our approach can be readily generalized to intepret experimental data in other cases.

## Introduction

The development and maintenance of tissues in multicellular organisms requires a diverse array of structural elements that link cells to each other and to the extracellular matrix [1, 2]. For epithelial tissues the main players in cell-cell adhesion are the proteins of the adherens junction complex: transmembrane cadherins and their binding partners that connect the actin cytoskletons of neighboring cells. To understand both healthy tissue architecture and abnormalities that lead to weakening of adhesion in epithelial tumors [3], it is necessary to decipher the underlying molecular mechanisms that regulate the stability of the junctions. Identifying the binding partners of cadherin, their functional roles and interplay under varying environmental conditions, has been a major research goal over the last three decades [2].

**Fig 1:**
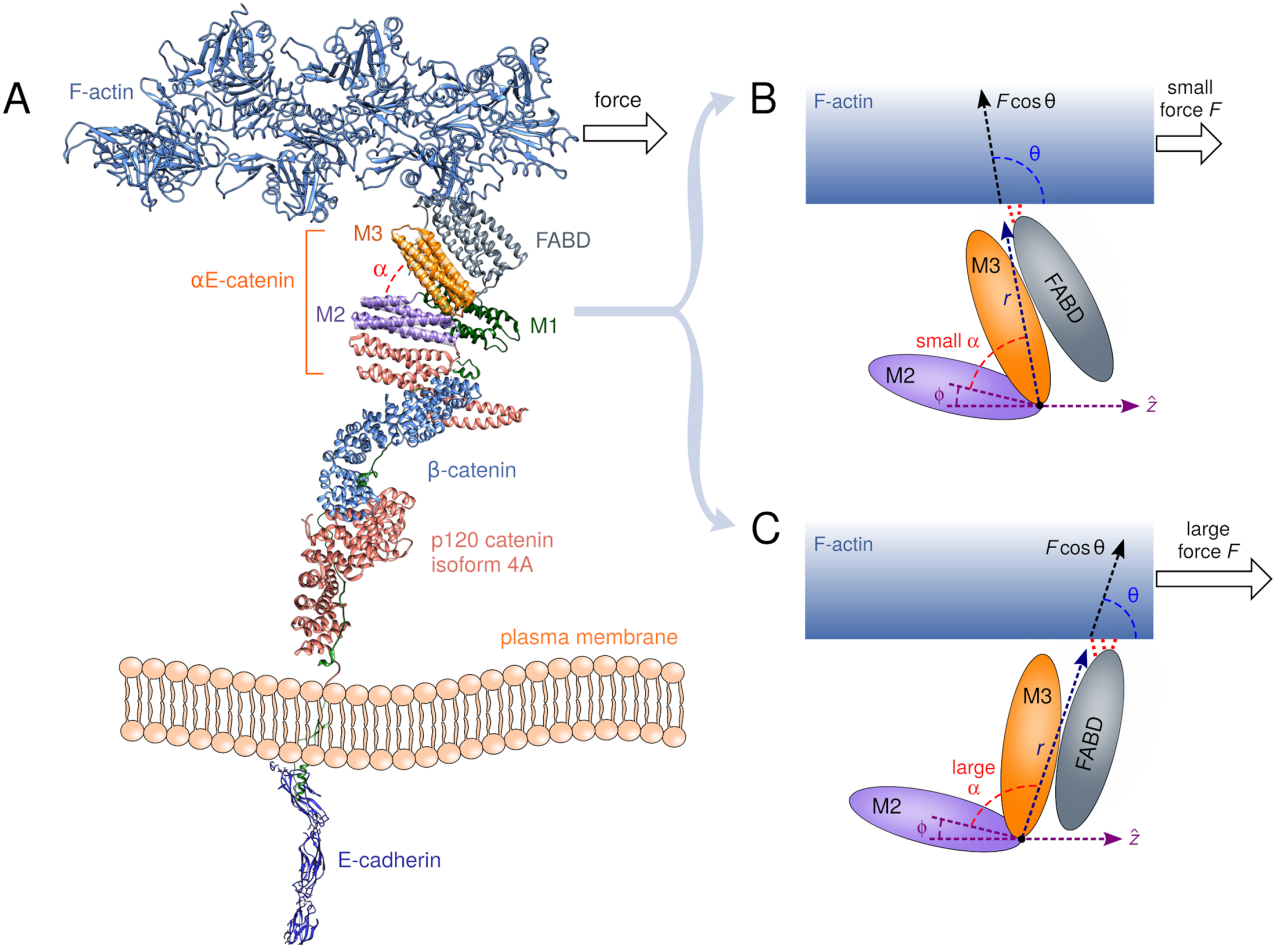
A schematic diagram showing hypothetical conformational changes of the cadherin-catenin-actin complex under force. A) A cartoon of the complex. In the absence of a crystal structure of the entirety, the diagram is drawn from the following PDB structures of various components: 3Q2V (E-cadherin), 3L6X (p120 catenin), 1I7W (*β*-catenin), 4IGG (*α*E-catenin), 1M8Q (F-actin). The arrangement of the structures relative to one another is a guess for the purposes of illustration. The theoretical model described in the text is independent of the details of this arrangement. B) The M region of *α*E-catenin, showing a conformation with small angle *α* between the M2 and M3 domains, favored at lower forces. The interactions (red dashed lines) between the adjacent F-actin binding domain (FABD) and F-actin depend on the conformational state of *α*E-catenin. C) Same as B, but in the large angle conformation, favored at larger forces. This results in an enhancement of FABD-actin interactions, leading to catch bond behavior.

The great challenge in achieving this goal is that binding between proteins is not a simple sum of pairwise interactions: the strength of adhesion between any two partners can be allosterically regulated by the presence or absence of other proteins in the complex, as well as conformational changes induced by external factors like mechanical tension [4]. For example, early studies established that the cytoplasmic domain of E-cadherin can bind to *β*-catenin [5, 6], and *β*-catenin can in turn bind to *α*E-catenin [7]. Since the latter was known to independently bind F-actin [8], naively one would assume that *α*E-catenin would be the bridge linking E-cadherin/*β*-catenin to F-actin, forming a minimal recipe for an adherens junction complex (see the schematic model in Fig 1). However subsequent *in vitro* experiments with purified proteins cast doubts on this model, showing that while E-cadherin/*β*-catenin/*α*E-catenin formed a stable complex, it had significantly lower affinity for F-actin than *α*E-catenin alone [9, 10].

This puzzling result was only clarified three years ago, when Buckley *et al.* added one more ingredient into the mix: applying physiological (pN-level) forces to the entire cadherin-catenin-actin (CCA) system in an optical tweezer [11]. Such external forces mimic the mechanical loads which the complex would feel *in vivo*, and thus would be a more realistic context to study complex formation than the earlier experiments in the absence of load. The results were dramatic: the mean lifetime of the CCA complex increased by a factor of 20 as force was increased from 0 to 10 pN, an unusual force-induced strengthening known as catch bonding [12]. The lifetime then fell off exponentially at higher forces, the conventional slip bond decay expected for most biological bonds under tension. The minimal CCA model of the adherens junction gained a new dimension of dynamic complexity: under the right amount of external load, the bond with actin is stabilized up to lifetimes of ∼1 s, perhaps long enough for vinculin, an additional binding partner between *α*E-catenin and actin to attach and strengthen the junction [13, 14].

Catch bonding has now been observed in a variety of adhesion and receptor proteins complexed with particular ligands, among them selectins [12], integrins [15], bacterial FimH [16], and the *αβ* T-cell receptor [17]. The phenomenon is not limited to protein-ligand complexes, but can occur even in single knotted proteins [18], *α*-helices [19], and force-sensitive functional groups in polymeric materials [20]. One of the most recent observations has been in vinculin [14] binding to actin, where the degree of strengthening under load also depends on the direction of the force. While all these examples highlight the crucial role of tension in regulating interactions, many of them also share the common feature that the structural and energetic details of how this regulation occurs at the molecular level remain largely a mystery. The force spectroscopy experiments that demonstrate protein-ligand catch bonding reveal only the distributions of unbinding times at different forces. We know from very general theoretical considerations that the underlying free energy landscape of a catch bond must necessarily be complex: a simple landscape with a single bound state energy well, and an end-to-end extension that increases monotonically with force, will always yield slip bond behavior [21]. Thus the most likely scenario for catch bonding is a landscape with heterogeneous bound states [22], corresponding to different molecular conformations that can dynamically interconvert under force. But for any specific catch bond system, like CCA, this hypothesis leads to a host of difficult questions: what are the structural differences between the different conformational states? What are the energy barriers between those states? For each state, what are the associated changes in the interaction energies at the bond interface, which are ultimately responsible for the catch bond behavior?

Modeling can assist in tackling these issues, but all current theoretical approaches, despite their various strengths, fall short of being able to directly answer the above questions. The most widely used descriptions of catch bonds are phenomenological [23–27], typically based on a kinetic network of strongly and weakly bound states [24, 28], with force biasing the system toward the strong state. While these models can fit experimental data and capture the essential conceptual basis of catch bonding—conformational heterogeneity—they are expressed in terms of transition rates between states. There is no direct connection between the fitted parameters and the structural features of those states, no way of estimating energy barriers, and no ability to rationalize or predict the results of mutation experiments on the bond lifetimes. Atomistic molecular dynamics simulations give important structural insights [29–32], but have their own limitations: conformational transitions and bond breaking in adhesion complexes at physiological forces typically occur on timescales (ms—s) many orders of magnitude larger than those accessible by all-atom simulations, precluding direct comparison to force spectroscopy experiments. Thus a compromise is needed, an approach that is able to fit experimental data, but with results that also have a concrete structural interpretation.

A recent study on the catch bonding in P- and L-selectin adhesion proteins pointed to a possible solution to this problem, introducing a novel, structure-based theory [33]. It provided an analytically solvable model for the mean bond lifetime, whose parameters could be directly linked to the energetics of the interface between the selectin protein and its ligand, as well as structural length scales in the complex. All the fitted parameters were physically reasonable, and in particular the extracted energies were consistent with available crystal structure data on the hydrogen bonding network at the interface. Such a model could for the first time rationalize how particular interfacial energy changes due to mutations would affect the observable bond dynamics. Unfortunately even this approach has an important shortcoming: it assumes the structural transition that occurs under force (in this case the rotation of two selectin domains with respect to each other) does not involve a significant energy barrier. In other words, the transition occurs on timescales much shorter than the mean bond lifetime. At any given force, the model thus yields a probability distribution of lifetimes (also known as a bond survival probability) that is single-exponential.

While the selectin-ligand and other systems [25, 34, 35] considered in Ref. [33] do exhibit single-exponential survival probabilities experimentally, the majority of adhesion systems where data is available do not, including CCA [11, 15, 36–39]. Thus there is a need for a model that is structure-based, analytically tractable, and which can account for the full complexity of bond survival probabilities observed empirically. The theory developed in the current work fulfills all these criteria. It reproduces the experimental lifetime distributions of CCA, and also links them to existing structural information on the conformations of *α*E-catenin. It provides the first estimates of the energy barrier height between these conformations as the complex remodels under force, as well as the resulting energetic changes at the actin interface. These predictions allow us to suggest a future set of experiments to validate the model. They also give insights into the role of the catenin energy barrier in physiological contexts, where a specific conformation of CCA may be required for efficient binding of vinculin to further stabilize the complex [14]. While our focus is on a single system, the theory framework itself is quite general, and can be be readily adapted to other cases. It subsumes earlier models of bond dynamics as special cases in certain limits, including both the barrier-less selectin model and the conventional Bell model for slip bonds. It thus has the potential to provide a unified analytical formalism for interpreting data from the entire spectrum of force-regulated adhesion complexes seen in nature.

## Methods

### Structure-based model

The key structural hypothesis underlying our theory is that conformational changes in the CCA complex induced by force allosterically regulate the interaction strength between F-actin and the C-terminal F-actin binding domain (FABD) [8] of *α*E-catenin (see schematic model in Fig 1). In the absence of a crystal structure of the FABD-actin interface, many questions remain about its molecular details [40, 41], and among the goals of our approach is to elucidate the overall actin-FABD bond energy and how it varies between different CCA conformations. The precise nature of the conformational changes that occur under tension is also not definitively established, though various lines of evidence point to the central role played by *α*E-catenin as the force transducer [42, 43], including recent dynamic FRET visualization of reversible conformational changes in the central domains of *α*E-catenin in a CCA complex under tension in living cells [44]. Fragmentary crystal structures of these central domains [43] suggest the potential of two alpha-helical bundles known as M2 and M3 (residues 396-506 and 507-631 respectively) to adopt different angles with respect to each other. The angle between the bundles (denoted by *α* in Fig 1) is likely to alter under applied tension, and thus the rotation of M3 with respect to M2 is a natural candidate for the main force-sensitive conformational change [32, 43]. For a catch bond to exist, conformations with small *α* should be associated with weaker FABD-actin binding, and those with larger *α* with stronger FABD-actin binding. As applied tension biases the system toward the latter conformations, this will lead to a regime where the effective bond lifetime increases with force. This rotation mechanism of catch-bond formation, where the relative orientation between two protein domains is coupled to the bond strength, has proven successful in explaining both experimentally and theoretically the catch bonds in several selectin systems [33, 45], and has recently been suggested as the underlying mechanism in catch bonds between the Notch receptor and certain ligands [36]. One important complication for *α*E-catenin, not present in the selectin cases, is the existence of a significant energy barrier to rotation: crystal structures [41, 43] and molecular dynamics simulations [32] highlight a number of salt bridges among the M-domains that stabilize the small-*α* orientation of M2 and M3. This will prove a crucial ingredient in explaining the dynamics and functional role of the bond, as we will discuss in more detail later.

Synthesizing all these structural considerations into an analytically tractable model, we will posit a minimal Hamiltonian *U*(*r*, *θ*) for the FABD-actin bond. The conformation-dependence of the bond is encoded in two structural variables (see Fig 1): i) the magnitude *r* = |**r**| of the vector **r** between the rotation pivot point (i.e. the junction of the M2 and M3 domains) and the FABD-actin interface; ii) the angle *θ* between **r** and the applied force $F\hat{z}$ acting on the bond through the actin. The overall geometry of *α*E-catenin relative to actin in Fig 1 mimics the optical tweezer experimental setup of Ref. [11], whose bond lifetime results we will analyze. That setup was in turn inspired by electron tomographic images showing the organization of actin filaments near the membrane relative to CCA complexes. Fixing $\hat{z}$ as the actin direction, the M2 domain could have an offset angle *ϕ* relative to **z**, making the relationship between the M2-M3 domain angle *α* and *θ* have the form: *α* = *π* − *θ* − *ϕ*. (As discussed in the next section on parameter estimation, the Ref. [11] experimental data was consistent with *ϕ* ≈ 0° to within a few degrees.) Because of steric effects between the domains and the nature of their junction, we assume the angle *α* can only take on values in some range *α*_min_ ≤ *α* ≤ *α*_max_, which means *θ* is restricted to the corresponding range *θ*_max_ ≥ *θ* ≥ *θ*_min_, where *θ*_max(min)_ ≡ *π* − *α*_min(max)_ − *ϕ*. The Hamiltonian *U*(*r*, *θ*) has the form:

$$\begin{array}{c} U\left(r,\theta \right)=\frac{1}{2}k\left(\theta \right){\left(r-r_{0}\right)}^{2}-Fr\text{cos}\theta +C\left(\theta \right) \end{array}$$ (1)

where

$$\begin{array}{c} \begin{array}{cc} k(\theta ) & =k_{0}+k_{1}\left(1+\text{cos}\theta \right),\\ C(\theta ) & =\{\begin{array}{cc} \frac{H(\text{cos}\theta -\text{cos}\theta_{\text{max}})}{\text{cos}\theta_{c}-\text{cos}\theta_{\text{max}}}, & \theta \geq \theta_{c}\\ \frac{\left(H-G\right)(\text{cos}\theta -\text{cos}\theta_{\text{min}})}{\text{cos}\theta_{c}-\text{cos}\theta_{\text{min}}}+G, & \theta <\theta_{c} \end{array}. \end{array} \end{array}$$ (2)

Let us consider each of the terms in Eq (1) in turn. The first term in the Hamiltonian *U* is an effective bond elastic energy with angle-dependent spring constant *k*(*θ*) and natural bond length *r*_0_. The distance *r* serves as an effective reaction coordinate for the bond, with bond rupture occurring if *r* > *r*_0_ + *d*, where *d* is the transition state distance. Thus the free energy barrier to bond rupture is *k*(*θ*)*d*^2^/2, which depends on the conformation through *k*(*θ*). Any angular function *k*(*θ*) can be expanded in Legendre polynomials *P*_*l*_(cos *θ*), and for our purposes it is sufficient to keep the two lowest-order terms (*l* = 0, 1) in the expansion, *k*(*θ*) = *k*_0_ + *k*_1_(1 + cos *θ*), with coefficients *k*_0_, *k*_1_ > 0. This function describes the key feature of the allosteric coupling between the *α*E-catenin conformation and the bond strength: as *θ* decreases under force, *k*(*θ*) increases, leading to a higher energy barrier to rupture. The extent of the bond strengthening is determined by the magnitude of *k*_1_. In analyzing the bond energetics later, it will be useful to express the role of *k*_0_, *k*_1_ equivalently through two energy parameters *E*_0_, *E*_1_ that have simpler physical interpretations. *E*_0_ is the free energy barrier to rupture at *α* = *α*_min_ when *F* = 0, given by *E*_0_ = (*k*_0_ + *k*_1_(1 + cos *θ*_max_))*d*^2^/2, and *E*_0_ + *E*_1_ is the free energy barrier to rupture at *α* = *α*_max_ when *F* = 0. The difference in barrier heights from *α*_min_ to *α*_max_ (responsible for the bond strengthening) is *E*_1_ = *k*_1_(cos *θ*_min_ − cos *θ*_max_)*d*^2^/2.

**Fig 2:**
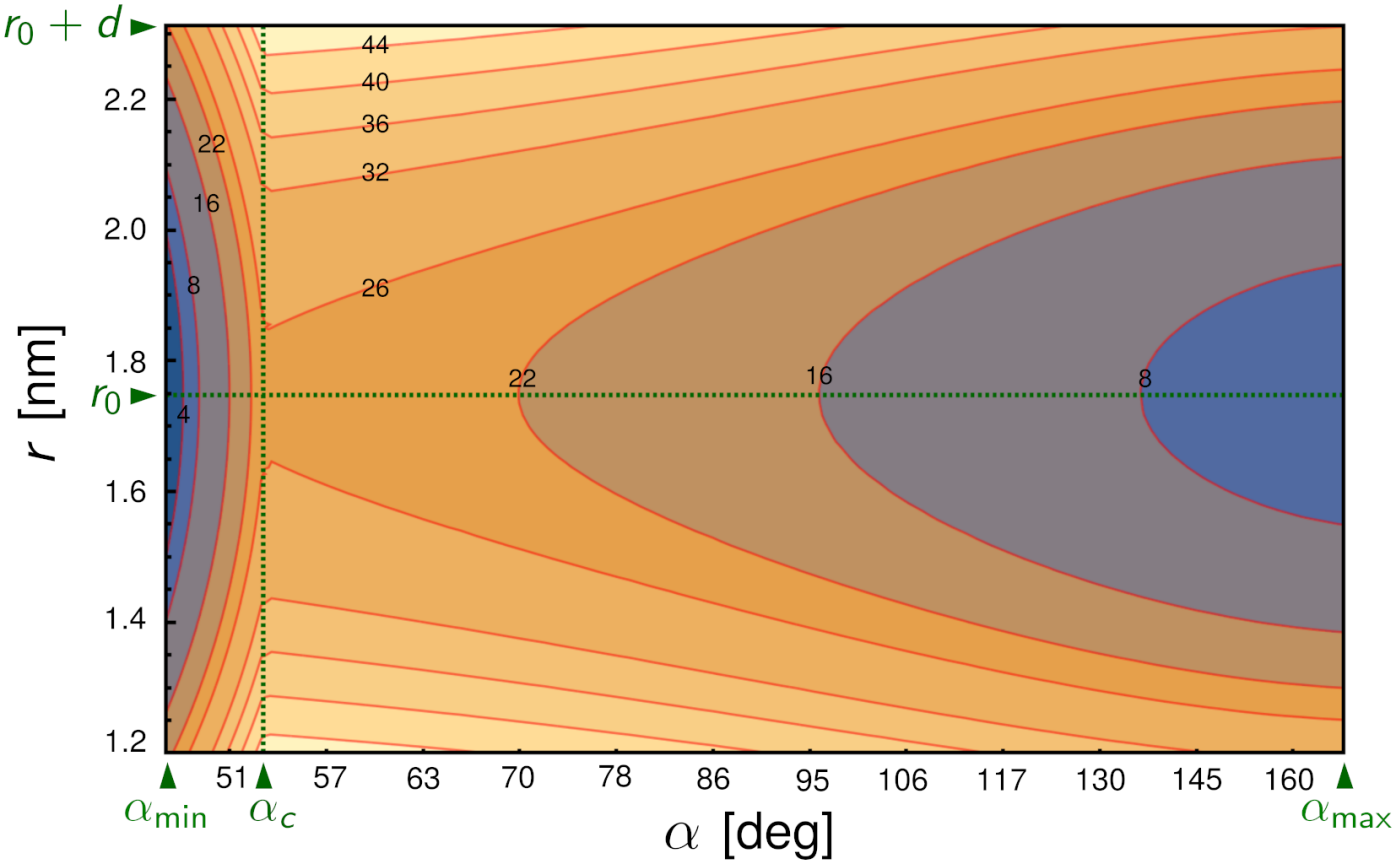
Energy landscape of the Hamiltonian *U* from Eqs (1) and (2) in terms of *r* and *α* = *π* − *θ* − *ϕ* at force *F* = 0, with the parameters given in Table 1 and described in the text. Energy contour labels are in units of *k*_*B*_*T*. The vertical dashed line corresponds to the transition angle *α*_*c*_, the horizontal dashed line to the natural bond length *r*_0_, and the top edge to the distance *r*_0_ + *d* beyond which the bond ruptures. The energy barriers to rupture are smaller in the region *α* ≤ *α*_*c*_ on the left, relative to the region *α* > *α*_*c*_ on the right. Since applied force *F* > 0 tilts the landscape toward larger inter-domain angles *α*, the mean bond lifetime will initially increase with force.

The second term in *U* describes the coupling of the Hamiltonian to the external applied force of magnitude *F*. It tilts the energy landscape toward larger *r* (increasing the chances of rupture at a given *θ*) and smaller *θ* (or equivalently larger *α*). The final term *C*(*θ*) in *U* describes a free energy barrier between the angular conformational states located at a particular transition angle *α*_*c*_ = *π* − *θ*_*c*_ − *ϕ*. This effectively subdivides the angular conformational space into two basins: a small inter-domain angle region (*α* ≤ *α*_*c*_ or *θ* ≥ *θ*_*c*_) and a large inter-domain angle region (*α* > *α*_*c*_ or *θ* < *θ*_*c*_). The barrier passing from small to large *α* has height *H*, and the barrier returning from large to small *α* has height *H*–*G*, with a possible free energy offset *G* between the two basins. As in the case of *k*(*θ*), we keep only terms up to linear order in cos *θ*, and make the barrier between the two regions cusp-like for analytical convenience. Using a more complicated form of *C*(*θ*), with a smooth rather than cusp-like barrier, would not significantly alter the results of the model (i.e. it would only lead to small corrections ∼*k*_*B*_*T* in the fitted results for the energy barriers, where *k*_*B*_ is the Boltzmann constant and *T* the temperature). A representative energy landscape for *U* at *F* = 0 is drawn in Fig 2 in terms of *r* and *α*, showing the two wells corresponding to the small *α* and large *α* conformational states.

The dynamics on this landscape is assumed to be described by diffusion of the vector **r** obeying a Fokker-Planck equation with potential *U* and diffusivity *D* = *k*_*B*_*T*/6*πηr*_0_, since the motion corresponds to a rearrangement of a protein domain with characteristic size *r*_0_. Here *η* is the viscosity of water, and for simplicity we ignore any prefactor due to the details of the domain shape in the diffusivity. The corrections due to such a prefactor are small, since it contributes only logarithmically to the fitted energies [33]. Reflecting boundary conditions are assumed at *θ*_min_ and *θ*_max_. The two main dynamical quantities of experimental interest are: (i) the mean bond lifetime *τ*(*F*), defined as the average time it takes to reach bond rupture, *r* = *r*_0_ + *d*, after the onset of an applied force of magnitude *F*. Prior to the force onset, the system is assumed to have equilibrated at zero force, in accordance with the experimental analysis in Ref. [11]; (ii) the survival probability distribution Σ_*F*_(*t*), defined as the probability that a bond has not yet ruptured by time *t* for a given *F*. The two quantities are related through $\tau \left(F\right)=\int_{0}^{\infty }dt\;\Sigma_{F}\left(t\right)$.

Calculating either *τ*(*F*) or Σ_*F*_(*t*) analytically is non-trivial for a multi-dimensional potential like *U*, but we can take advantage of the double-well structure of the energy landscape. As shown in detail in the S1 Appendix, we first find approximate analytical expressions for four individual transition rates: crossing the barrier from the small to large *α* well, the reverse transition, bond rupture directly from the small *α* well, and bond rupture directly from the large *α* well. We then combine these expressions into analytical results for *τ*(*F*) and Σ_*F*_(*t*) in terms of the Hamiltonian parameters.

The final expressions for *τ*(*F*) and Σ_*F*_(*t*) in the S1 Appendix are rather complex. But as described in the next section, Σ_*F*_(*t*) can be readily incorporated into a maximum likelihood estimation approach to find best-fit Hamiltonian parameters given an experimental data set, i.e. measurements of bond lifetimes at various forces. Moreover *τ*(*F*) reduces to earlier, simpler models of bond dynamics in certain limits. When *H* = *G* = 0, *θ*_min_ = 0, *θ*_max_ = *π*, we exactly recover the expression for *τ*(*F*) in the absence of an angular barrier (and a corresponding Σ_*F*_(*t*) which is approximately single-exponential), used to describe selectin-ligand catch bonds in Ref. [33] (see details in the S1 Appendix). If in addition we set *k*_1_ = 0, so that *k*(*θ*) = *k*_0_ becomes independent of *θ*, we do not have any force-enhancement of the bond lifetime. In this limit *τ*(*F*) ∝ exp(−*Fd*/*k*_*B*_ *T*), the classic Bell model for conventional slip bonds [46]. The fact that we can smoothly interpolate between different regimes in parameter space, describing qualitatively different modes of force regulation, is one of the strengths of our approach. This allows us, for example, to make predictions for possible mutation experiments that alter the system parameters, and see to what extent the dynamics are robust to such changes.

## Results

### Maximum likelihood estimation of the model parameters from force spectroscopy data

To estimate the Hamiltonian parameters and gain insights into the structural mechanisms of catch bonding in the CCA complex, we fit the model to the raw data from the optical tweezer force spectroscopy experiment in Ref. [11]. This data consists of 803 measurements of the bond lifetime under varying force conditions from *F* = 0.7–33 pN, the same dataset whose histogram is depicted in Fig. 4A of Ref. [11]. For a given parameter set and force *F*, the probability to observe a bond lifetime between *t* and *t* + *dt* is −*dt* *d*Σ_*F*_(*t*)/*dt*. We could thus construct an overall likelihood function for the data set given the parameters (details in the S1 Appendix), and maximize it to find the best estimate for the parameters.

For numerical convenience, it was useful to do the fitting in two stages: in the first stage we fixed values for the minimum M2-M3 inter-domain angle *α*_min_ and angle offset *ϕ*, and then maximized the likelihood function over the remaining parameters for these fixed values. In the second stage we then repeated this procedure for different choices of *α*_min_ and *ϕ*, to find the overall optimum. The largest likelihoods occurred in the range *α*_min_ = 40–50° and *ϕ* = −5 to 5°, yielding results for the remaining parameters identical to within error bars. The best-fit values reported in Table 1 are for *α*_min_ = 48° and *ϕ* = 0°.

**Table 1:** Model parameters. Parentheses after the values denote the uncertainty in the last digit.

	Physical interpretation	Value
*E*_0_	Free energy barrier to rupture at small *α*	21.8(2) *k*_*B*_*T*
*E*_1_	Extra free energy barrier to rupture at large *α*	5.8(2) *k*_*B*_*T*
*H*	Free energy barrier for small-to-large *α* transition	25(2) *k*_*B*_*T*
*G*	Free energy offset between angular basins	5(2) *k*_*B*_*T*
*d*	Transition state distance	0.56(1) nm
*r*_0_	Distance from hinge to FABD-actin interface	1.7(2) nm
*α*_min_	Minimum angle between M2-M3 domains	48°
*α*_*c*_	Transition angle between small and large *α* wells	53(3)°
*α*_max_	Maximum angle between M2-M3 domains	169(9)°

The predicted *α*_min_ range is consistent with available structural information. Though the experiment [11] was done using monomeric zebrafish *α*E-catenin, for which there is no crystal structure, we can compare to known homologous structures from other species and computational structure prediction results. 47° was the smallest angle observed in an analysis of available crystal structure fragments of the M2-M3 domains from mouse and human *α*E-catenin [43], and 48° is the M2-M3 angle observed in the individual monomers of the full-length human *α*E-catenin homodimer (PDB: 4IGG) [41]. Plugging the zebrafish *α*E-catenin sequence into the I-TASSER structure prediction server [47, 48] yields an M2-M3 angle of 45 ± 1° among the five best structures.

**Fig 3:**
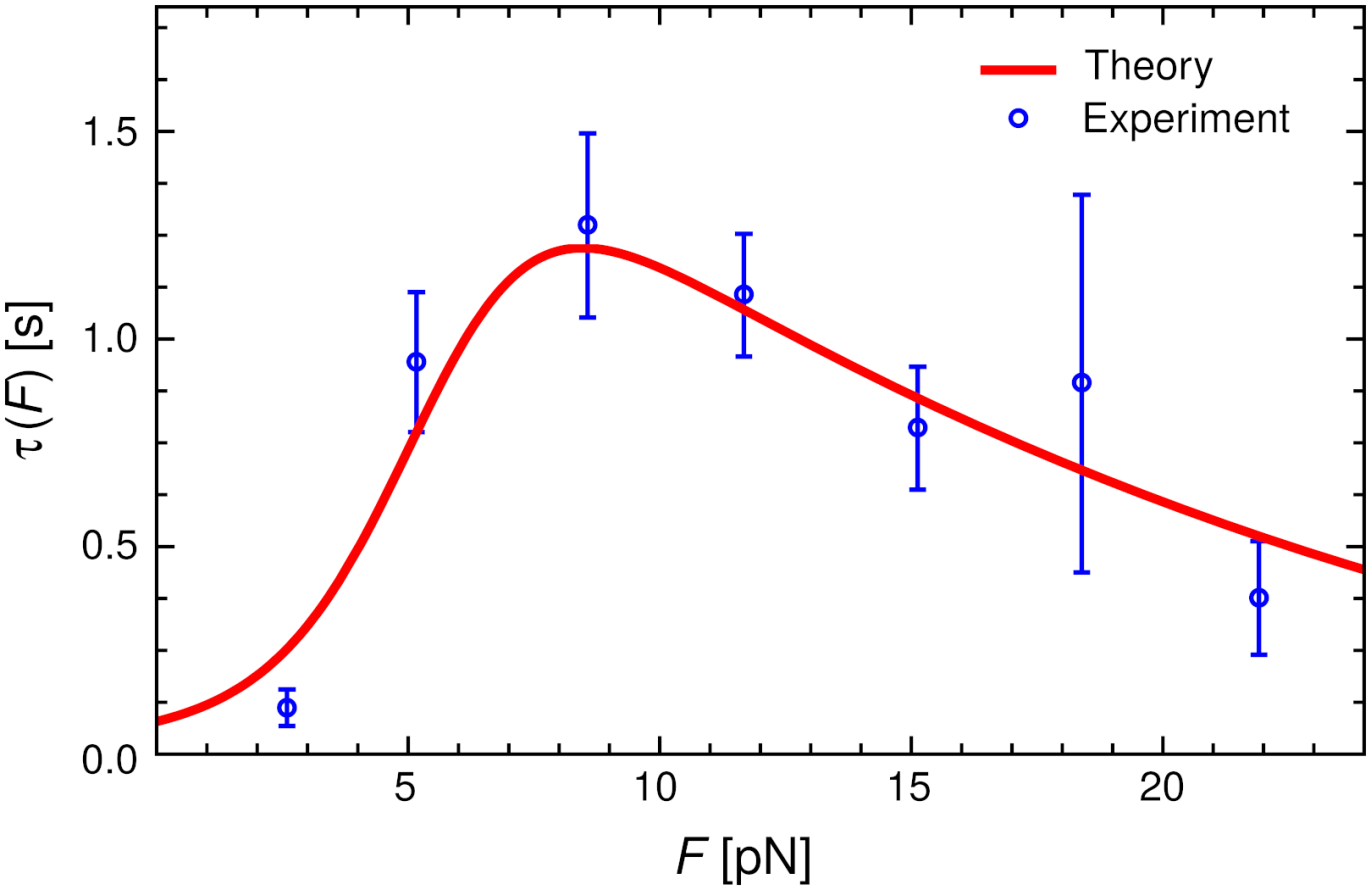
Experimental mean bond lifetime *τ*(*F*) versus force *F* (symbols) from Ref. [11] compared to the theoretical model with best-fit parameters from Table 1 (curve).

**Fig 4:**
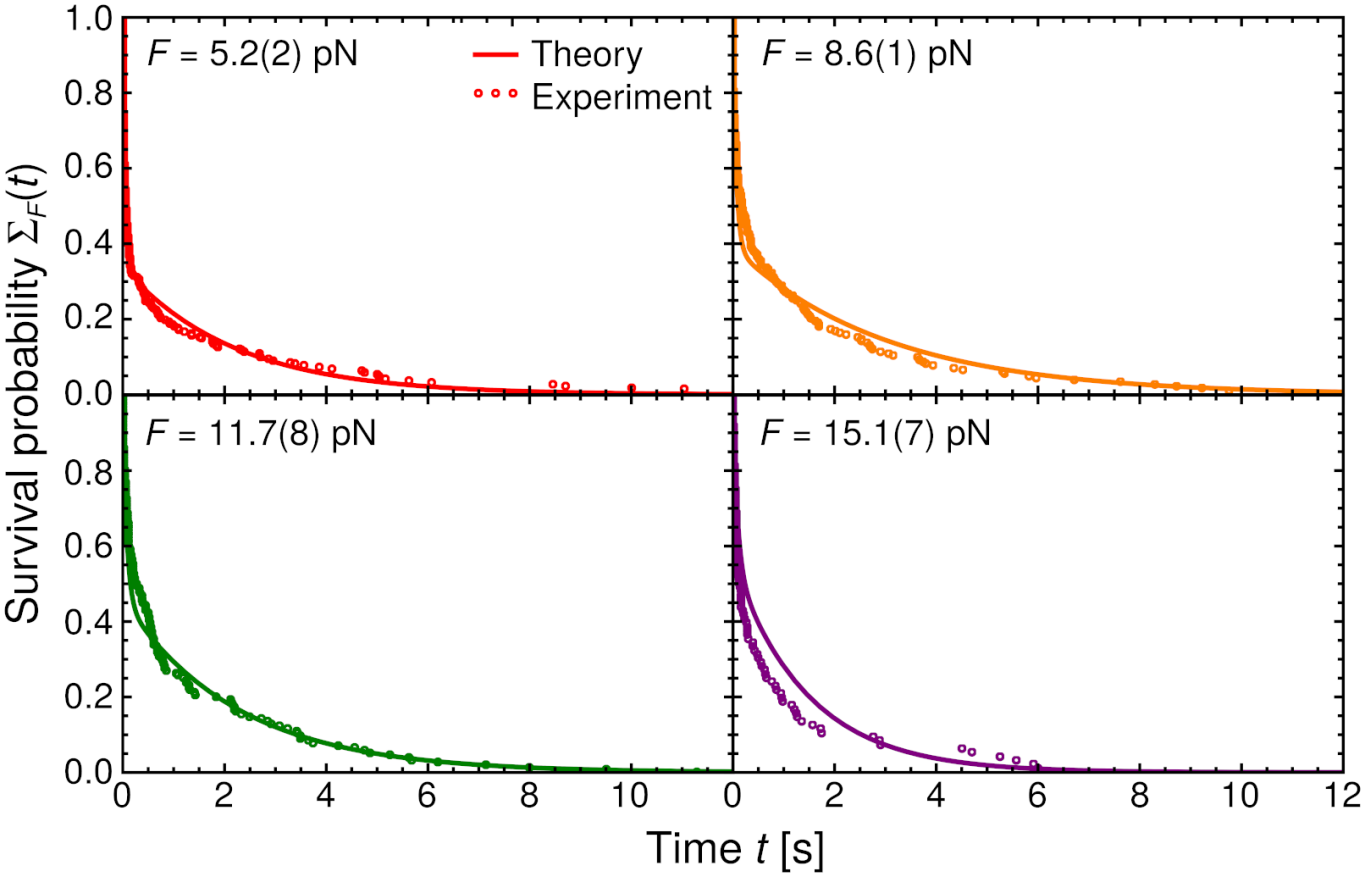
Bond survival probability Σ_*F*_(*t*) versus time *t* for four different forces *F*. Theory results are shown as curves, and the corresponding experimental data [11] as symbols.

The theoretical mean bond lifetime *τ*(*F*) is compared to the experimental results from Ref. [11] in Fig 3, and the analogous comparison for the survival probabilities Σ_*F*_(*t*) at different *F* is shown in Fig 4. The agreement between theory and experiment is excellent, with the model capturing not only the catch bond trend in *τ*(*F*), but also the clear double-exponential behavior in Σ_*F*_(*t*). As we will discuss in more detail below, the observation of two exponential regimes is closely connected to the presence of a significant energy barrier between the small *α* and large *α* conformations.

### Interpretation of the model parameters, and corroboration from structural data

The value of the model comes not just from the fact that it can fit the experimental data, but that its parameters have a direct physical interpretation that illuminates the structural mechanism of the CCA catch bond. The energy barrier at the transition angle *α*_*c*_ = 53° divides the parameter space into two basins: a narrow basin between *α*_min_ = 48° to *α*_*c*_, and a much wider basin between *α*_*c*_ and *α*_max_ = 169°. The narrow range suggests the M3 domain is held rigidly in place relative to M2 in the small *α* case, with limited rotational mobility, but once the stabilizing interactions at the hinge between M2 and M3 are broken, M3 can swing out to a larger angle. Of course the idea of solid body rotation about a hinge is a simplification: the protein domains are plastic objects that can continuously deform under tension, but picturing an overall rotation is still a useful first approximation. The parameter *r*_0_ = 1.7 nm, the distance between the hinge and the FABD-actin interface in the simple picture, can more accurately be interpreted as the effective size of the protein regions undergoing reorientation under force.

**Fig 5:**
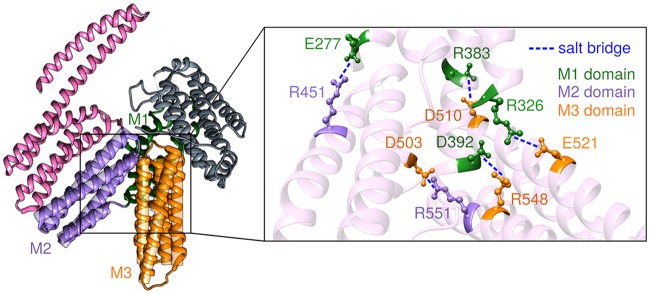
The salt bridge network in the hinge region between the M1, M2, and M3 domains of *α*E-catenin (PDB: 4IGG) [41].

The strength of the interactions in the hinge region is reflected in the angular energy barrier height *H* = 25 *k*_*B*_*T*, whose full significance we will explore below. The existence of this barrier is supported by corroborating evidence from a crystal structure [41] of *α*E-catenin (PDB: 4IGG), which shows five inter-domain salt bridges in the hinge region where the M1, M2, and M3 domains meet (Fig 5). If each salt bridge roughly contributes 4–8 *k*_*B*_*T* to the overall barrier [49], this is consistent with the magnitude of *H*. Molecular dynamics simulations also point to the stabilizing role of the salt bridges. Li *et al.* [32] compared trajectories measuring the M2-M3 angle for the wild-type structure, initially starting in the small *α* state, to trajectories of mutants where one of the salt bridges is disrupted (i.e. E521A or R551A). The latter show the system venturing more readily to larger angles relative to the wild-type, as expected for a smaller barrier *H*.

Having two conformational states at small and large *α* in itself does not guarantee catch bond behavior. What leads to lifetime enhancement under force is the fact that these states are allosterically coupled to the strength of the FABD-actin bond, which changes from *E*_0_ = 21.8 *k*_*B*_*T* at small *α* to *E*_0_ + *E*_1_ = 27.6 *k*_*B*_*T* at large *α*. Though we do not have any crystal structure of the FABD-actin interface, it is instructive to compare the value of *E*_0_ + *E*_1_ to a different catch bond system: the P-selectin complex with the ligand PSGL-1, where *E*_0_ + *E*_1_ = 27 *k*_*B*_*T* [33] in the extended state favored at larger forces. The peak bond lifetime in P-selectin/PSGL-1 (∼1.1 s) is also very similar to CCA (∼1.1 s in Fig 3). Conveniently we do have the crystal structure of P-selectin-PSGL-1 in the extended conformation (PDB: 1G1S) [50], showing that 20 hydrogen bonds contribute to *E*_0_ + *E*_1_, consistent with a contribution of 1.2–1.5 *k*_*B*_*T* per hydrogen bond, typical for hydrogen bonds in proteins [51]. We thus predict a similar number of hydrogen bonds at the FABD-actin interface in the large angle state (or fewer if salt bridges are involved). The allosteric change between the angular states translates into an interface energy difference of *E*_1_ = 5.8 *k*_*B*_*T*, about 4-5 hydrogen bonds or one salt bridge.

The energy offset parameter *G* = 5 *k*_*B*_*T* plays the important role of biasing the system toward small *α* when the force is small. The equilibrium probability $p_{\text{S}}^{0}$ of having *α* < *α*_*c*_ at *F* = 0 is $p_{\text{S}}^{0}=0.77$ (see S1 Appendix for the derivation). As *F* is increased, the energy landscape is tilted toward higher *α*, and the barrier to FABD-actin bond rupture shifts from *E*_0_ to *E*_0_ + *E*_1_, causing the lifetime enhancement. But the fact that the system is equilibrated at *F* = 0 before the application of force means that both large and small angle conformations are initially populated. The significant angular barrier *H* and the finite bond lifetime means that these populations do not necessarily have a chance to fully re-equilibrate once *F* > 0 is applied, during the time before rupture occurs.

These two populations, one with a smaller barrier to rupture than the other, explain the distinct double-exponential behavior of Σ_*F*_(*t*) (Fig 4; see also Fig. S2C of the SI). To understand this more concretely, a useful quantity is the probability of being in the small *α* state at the moment of rupture, the so-called splitting probability *π*_S_ (details given in the S1 Appendix). In the hypothetical scenario of arbitrarily long-lived bonds, where there is time for many transitions between the small and large *α* states, *π*_S_ ≈ *p*_S_, the equilibrium probability of being in the small *α* state. But in many cases the bond lifetime is too short for equilibration, and *π*_S_ may be very different from *p*_S_. For example at *F* = 15.1 pN (the last panel in Fig 4), *p*_S_ = 10^−4^, but *π*_S_ = 0.47. The tiny value of *p*_S_ means that, given enough time, the initial fraction, $p_{\text{S}}^{0}=0.77$, of systems that start at small *α* should eventually transition to the large *α* state preferred at high forces, and almost never return. If that were actually the case, the survival probability at *F* = 15.1 pN would have been to very good approximation a single exponential, since rupture would occur almost entirely from the large *α* state. In reality, because of the barrier *H* slowing down angular transitions, the majority of those small *α* systems do not have enough time to transition. They thus stay in the small *α* state until rupture, giving a sizable *π*_S_. This leads to a short lifetime exponential regime in Σ_*F*_(*t*), in addition to the longer lifetime exponential regime corresponding to ruptures from large *α*.

The final parameter in the model, the transition state distance *d* = 0.56 nm, represents how much the FABD-actin bond interface can be deformed before rupture. The value is within the range expected of most proteins (<2 nm) [52]. Putting everything together, we thus see that the fitted model parameters are all within physically realistic ranges, and consistent with all the available evidence both from the Buckley *et al.* experiment and earlier studies.

## Discussion

### Mutations to the angular barrier *H*, and its potential biological role

Disrupting the stability of the hinge region (Fig 5) with mutations at the M2-M3 interface (R551A) or M1-M3 interface (E521A) has been experimentally investigated to probe the role of the hinge in vinculin binding [43]. The underlying presumption is that the large *α* conformation, which is more accessible when the hinge is destabilized, exposes the vinculin binding site in the M1 domain. This would explain the enhanced binding affinity of the R551A and E521A mutants to the D1 domain of vinculin seen in the experiments. Of course in nature, access to the large *α* conformation is controlled not by mutations to the hinge, but by application of force, leading to the speculation that the *α*E-catenin system acts like a force-dependent “switch” [43], with tension favoring a large *α* conformation, which in turn enhances both vinculin and *F*-actin bond strengths.

In the context of the model, there are two scenarios for what might occur when the salt-bridge network at the hinge is disrupted: (i) the angular barrier energy *H* is decreased, since this is the parameter most directly related to the stability of the hinge, but other parameters in the model remain unaffected; (ii) the decrease of *H* is allosterically coupled to changes in the FABD-actin interfaces energies *E*_0_, *E*_1_ or other structural parameters. The latter would be reminiscent of the case of L-selectin, where experimental mutations at the hinge between the lectin and EGF domains [25] led to allosteric changes in energies at the ligand-binding interface [33]. The possibility of scenario (ii) will have to await future experimental data, but we can explore scenario (i) theoretically. This also allows us to investigate the biological significance of the angular barrier *H*.

**Fig 6:**
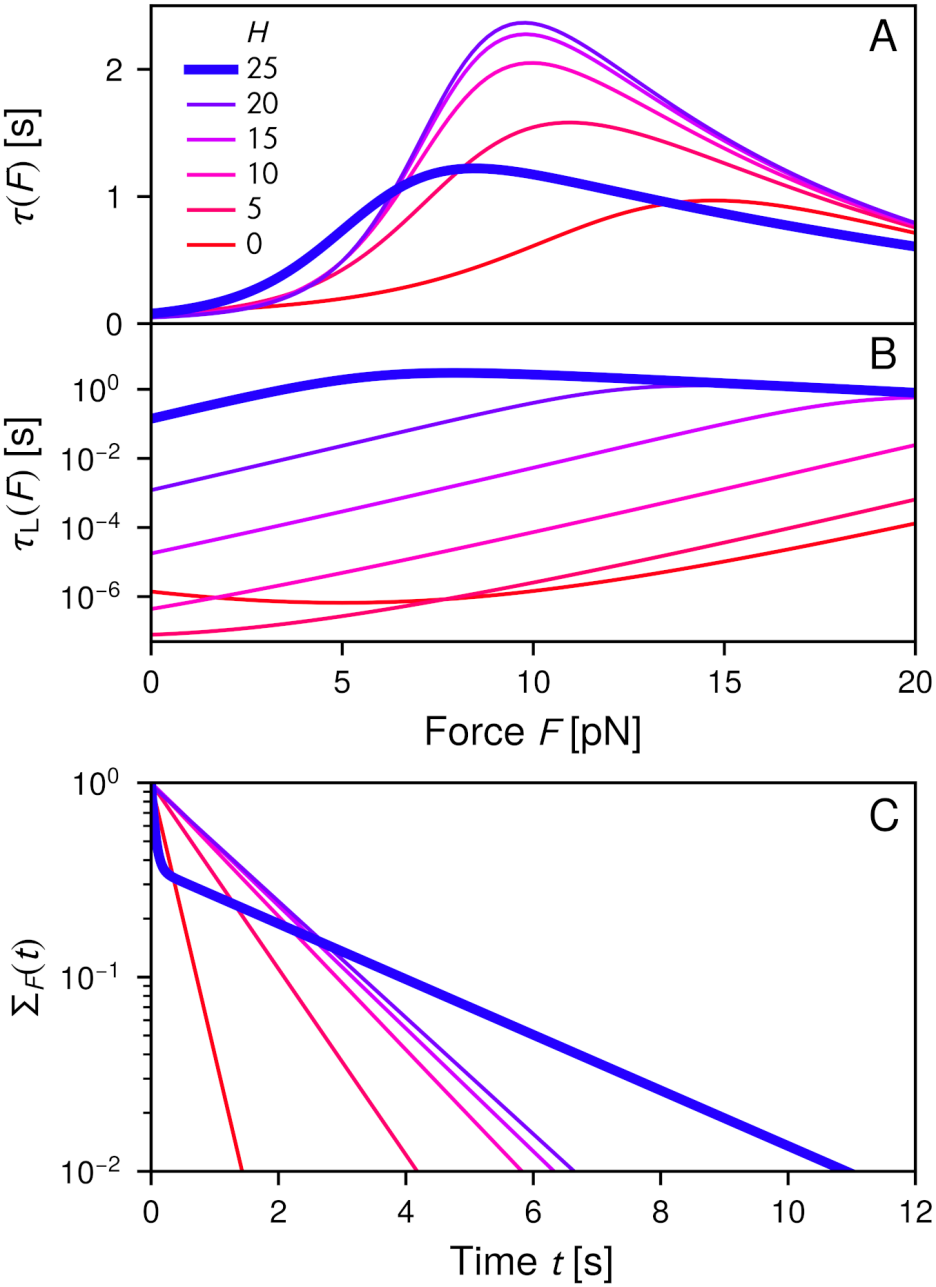
The effects of mutating the angular barrier height *H* from the original value of 25 *k*_*B*_*T* down to zero, in increments of 5 *k*_*B*_*T*, leaving all other model parameters fixed at their Table 1 values: A) the mean bond lifetime *τ*(*F*); B) the mean lifetime *τ*_L_(*F*) of remaining in the large angle conformational state, *α* > *α*_*c*_, measured from the initial time of entry into the state; C) the survival probability Σ_*F*_(*t*) at *F* = 7 pN.

Fig 6A shows what happens to the mean bond lifetime *τ*(*F*) when *H* is decreased from its wild-type value of 25 *k*_*B*_*T* in increments of 5 *k*_*B*_*T* (roughly corresponding to removal of individual salt bridges), while all other parameters are fixed at their Table 1 values. The catch bond behavior is preserved, but with opposite trends at small and large forces: at small forces *τ*(*F*) generally decreases with decreasing *H*, while at larger forces it initially increases by about a factor of two at the maximum, and then decreases gradually. These changes are due to the fact that transitions between the small and large *α* states become easier with decreasing barrier heights. At smaller forces, where the weaker small *α* states are preferred, some fraction of systems that would have ruptured from the stronger large *α* state can now transition to small *α* before rupturing. The converse is true at larger forces, where we now allow more small *α* states to transition to the preferred large *α* state before rupture.

Consistent with this, the lifetimes within each angular domain are drastically affected by the mutation. Fig 6B shows *τ*_L_(*F*), the mean duration of the large *α* state (from initial entry into the state until either rupture occurs or a transition to small *α*; see S1 Appendix for details). For the wild-type value *H* = 25 *k*_*B*_*T*, there is a broad force region, *F* ≈ 4–18 pN, where the large angle state survives for macroscopic times comparable to the maximum bond lifetime, *τ*_L_(*F*) > 1 s. When *H* = 20 *k*_*B*_*T* this region is decreased to *F* ≈ 12–18 pN, and then vanishes entirely at smaller *H*. With a decreasing barrier, the time spent at large *α* becomes significantly briefer, reduced by 4-6 orders of magnitude at *H* = 0. At *H* = 25 *k*_*B*_*T* a typical system trajectory may have involved zero or one transition across the angular barrier, and then rupture. In contrast at smaller *H* the system makes a large number of angular transitions before the bond breaks. The result is that the double-well nature of the energy landscape is averaged out, and the survival probability Σ_*F*_(*t*) switches from double-exponential at *H* = 25 *k*_*B*_*T* to mainly single-exponential at *H* ≤ 20 *k*_*B*_*T*, as seen in Fig 6C.

Thus while the presence of a large *H* barrier is not necessary for catch bonding, it is necessary to stabilize the large *α* conformational state so that it persists for long durations. A larger *τ*_L_(*F*) over a wide force range comes at the price of a somewhat smaller maximum *τ*(*F*). But this may be biologically preferred if the macroscopic duration of the large *α* state is necessary to allow time for additional binding partners (like vinculin) to dock before rupture or the transition to small *α*. Indeed two potentially fruitful future lines of experimental inquiry would be: a) to first study the CCA catch bond under different mutations to the *α*E-catenin hinge region. The mutations would have a clear signature of their effect on *H* by the change in the nature of the survival probability distribution [Fig 6C]. Whether the response of *τ*(*F*) would follow the trend in Fig 6A would determine if scenario (i) were true, or whether additional allosteric effects like in scenario (ii) are also present; b) to study the binding affinity or bond lifetime of vinculin to the CCA complex under these same mutations. This would elucidate whether the increased lifetime of the large *α* state, facilitated by the angular barrier, is also required for effective vinculin binding. One can also imagine an alternative vinculin binding mechanism like induced fit, where its affinity might be independent of the lifetimes or relative populations of the *α*E-catenin conformational states.

### Conclusions

The model presented here is the first quantitative, structural model for the catch bond in the cadherin-catenin-actin complex. It provides a full interpretation of the force spectroscopy data from the Buckley *et al.* experiment [11], highlighting the central role of *α*E-catenin as a force-transducing conformational switch [42–44]. The switch mechanism, based on small and large angle catenin conformations with different FABD-actin bond strengths, is to date the most plausible molecular explanation of the CCA catch bond. Force induces a small-to-large angle transition over a substantial energy barrier resulting from a network of salt bridges. This transition in turn allosterically modifies the strength of the catenin-actin interface, resulting in the catch bond behavior. The energy barrier, captured in the parameter *H* in our model, leads to the double-exponential survival probabilities seen experimentally. Additionally, once the system transitions to the large *α* conformation, the barrier allows it to remain there a significant fraction of the bond lifetime, perhaps facilitating the binding of other proteins like vinculin which play major roles in the physiological complex. While the model parameters are consistent with all the available evidence, including structural information about the *α*E-catenin hinge region, full corroboration of the mechanism will require further experiments to check whether alterations in the *α*E-catenin conformational stability have the posited effects on bond observables. Moreover, future crystal structures of the FABD-actin interface would allow verification of the *E*_0_ and *E*_0_ + *E*_1_ energy scales predicted by our approach.

Of course it is always possible that an alternative conformational mechanism will emerge for the CCA catch bond. Any competing explanation will still have to include a conformational change whose dynamics are slowed down by an energy barrier ≫ *k*_*B*_*T*, since this is the only way to have a catch bond with double-exponential survival probabilities. One of the attractive features of our model is that it can be readily adapted for such an eventuality. The current Hamiltonian is expressed in terms of bond distance and inter-domain angle, but analogous Hamiltonians can be formulated, replacing the angle with another conformational coordinate. The model can even generalize to more than two conformational basins in the energy landscape, separated by different barriers, if the structural evidence points in that direction. The basic approach stays the same, and analytical expressions for the bond lifetimes and distributions can always be derived to fit to experimental data. Given the ubiquity of multi-exponential lifetime distributions in catch bonding systems [11, 15, 36–39], implicating conformational transitions with non-trivial energy barriers, our approach thus might provide a universal framework for structural modeling of catch bonding. And it is not only limited to multi-exponential distributions, since single-exponential behaviors (for both catch and slip bonding) are just special cases of the model parameters. The usefulness of our theory starts at the cadherin-catenin-actin system, but hopefully will not end there.

## Supporting information

S1 Appendix Analytical derivations of main results and fitting details. (PDF)
