# Supplementary material for: Unraveling the mechanism of the cadherin-catenin-actin catch bond
Source: PLoS Comput Biol. 2018 Aug 17;14(8):e1006399. doi: 10.1371/journal.pcbi.1006399 (PMC6114904; doi:10.1371/journal.pcbi.1006399)
Supplement: S1 Appendix — (PDF) [file pcbi.1006399.s001.pdf]

S1 Appendix for
“Unraveling the mechanism of the cadherin-catenin-actin catch bond”

Shishir Adhikari, Jacob Moran, Christopher Weddle, and Michael Hinczewski
Department of Physics, Case Western Reserve University, Cleveland OH, 44106, U.S.A.

I. DERIVATION OF MEAN LIFETIME $\tau(F)$ AND SURVIVAL PROBABILITY $\Sigma_F(t)$

The following sections contain a full derivation of the main observable quantities of interest, the mean bond lifetime $\tau(F)$ and survival probability $\Sigma_F(t)$. Since the derivation involves a large number of individual components, Table S1 summarizes the main analytical quantities, their meaning, and the equations where they are defined.

Quantity	Meaning	Equation(s)
$U(r,\theta)$	bond Hamiltonian	(S1)-(S2)
$V(r,\theta)$	effective Fokker-Planck potential energy	(S6)
k_{10}	transition rate from state 1 to 0 (rupture)	(S15)-(S17)
k_{20}	transition rate from state 2 to 0 (rupture)	(S18)-(S19)
k_{12}	transition rate from state 1 to 2	(S26)-(S27)
k_{21}	transition rate from state 2 to 1	(S28)
τ_i	mean first passage time from state i to rupture	(S35)
p_i^0	probability of state i at time $t=0$	(S36)
τ	mean bond lifetime	(S37)
Σ_F	survival probability	(S38)-(S39)
π_S	probability of being in small α state at rupture	(S41)-(S42)
τ_L	mean duration of large α conformation (state 2)	(S43)

TABLE S1. Summary of main analytical results in the S1 Appendix, with corresponding equation numbers.

A. Fokker-Planck equation describing the bond dynamics

The theoretical model of the bond dynamics is based on diffusion of the bond vector $\mathbf{r} = (r,\theta,\phi)$ on an energy landscape defined by the Hamiltonian $U(r,\theta)$ in Eqs. 1-2 in the main text:

$$U(r,\theta) = \frac{1}{2}k(\theta)(r-r_0)^2 - Fr\cos\theta + C(\theta) \tag{S1}$$

where

$$k(\theta) = k_0 + k_1(1+\cos\theta),$$
$$C(\theta) = \begin{cases} \frac{H(\cos\theta-\cos\theta_{\max})}{\cos\theta_c-\cos\theta_{\max}}, & \theta\ge\theta_c \\ \frac{(H-G)(\cos\theta-\cos\theta_{\min})}{\cos\theta_c-\cos\theta_{\min}}+G, & \theta<\theta_c \end{cases}. \tag{S2}$$

To restrict the dynamics to the angular region $\theta_{\min}\le\theta\le\theta_{\max}$, we assume $U(r,\theta)=\infty$ for $\theta<\theta_{\min}$ and $\theta>\theta_{\max}$. Note that $U(r,\theta)$ depends on the applied force F on the system, so every observable derived from $U(r,\theta)$ below also implicitly depends on F, even if the dependence is not explicitly indicated in the notation.

Given a diffusivity $D = k_BT/6\pi\eta r_0$, the probability $\Psi(\mathbf{r},t)$ to find the system with vector $\mathbf{r}$ at time t obeys a Fokker-Planck equation in spherical coordinates of the form:

$$\frac{\partial\Psi}{\partial t} = \frac{D}{r^2}\frac{\partial}{\partial r}\left[r^2 e^{-U}\frac{\partial(e^U\Psi)}{\partial r}\right] + \frac{D}{r^2\sin\theta}\frac{\partial}{\partial\theta}\left[\sin\theta e^{-U}\frac{\partial(e^U\Psi)}{\partial\theta}\right] + \frac{D}{r^2\sin^2\theta}\frac{\partial}{\partial\phi}\left[e^{-U}\frac{\partial(e^U\Psi)}{\partial\phi}\right], \tag{S3}$$

Note that throughout the appendix we will work in units where $\beta=(k_BT)^{-1}=1$, so that all energies are effectively measured in units of k_BT. Since $U(r,\theta)$ is independent of ϕ, we can define a marginal probability $P(r,\theta,t)$ by multiplying Ψ with the spherical Jacobian and integrating over the angle ϕ,

$$P(r,\theta,t) \equiv r^2\sin\theta\int_0^{2\pi} d\phi\,\Psi(\mathbf{r},t), \tag{S4}$$

allowing us to write Eq. S3 as a 2D Fokker-Planck equation in terms of $P(r,\theta,t)$,

$$\frac{\partial P}{\partial t} = D\frac{\partial}{\partial r}\left[e^{-V}\frac{\partial(e^V P)}{\partial r}\right] + \frac{D}{r^2}\frac{\partial}{\partial \theta}\left[e^{-V}\frac{\partial(e^V P)}{\partial \theta}\right]. \tag{S5}$$

Here $V(r,\theta)$ is an effective potential defined by

$$V(r,\theta) \equiv U(r,\theta) - k_B T \ln(r^2 \sin\theta). \tag{S6}$$

We will not be interested in solving Eq. (S5) directly, but rather answering a closely related question: the mean first passage time (MFPT) to escape from a region in parameter space. Consider a region R of the (r,θ) space, with boundary ∂R, and let us focus on some subset $A \subseteq \partial R$ of this boundary. Let $\tau_{RA}(r,\theta)$ denote the MFPT to any point on A, given that we started at some point (r,θ) in the interior of R at $t=0$. We assume we have chosen R such that there are reflecting boundaries on the portion of ∂R not in A, namely $U(r,\theta)=\infty$ for $(r,\theta) \in \partial R \setminus A$. This guarantees that $\tau_{RA}(r,\theta)$ is finite, and satisfies the backward Fokker-Planck equation [1],

$$D\frac{\partial}{\partial r}\left[e^{-V}\frac{\partial \tau_{RA}}{\partial r}\right] + \frac{D}{r^2}\frac{\partial}{\partial \theta}\left[e^{-V}\frac{\partial \tau_{RA}}{\partial \theta}\right] = -e^{-V}, \tag{S7}$$

with absorbing boundary conditions $\tau_{RA}(r,\theta)=0$ for $(r,\theta) \in A$. To directly solve for the main experimental observable of interest, the mean bond lifetime, we would set R to be the entire parameter space region where the bond is intact, $r < r_0 + d \equiv b$, $\theta_{\min} \le \theta \le \theta_{\max}$, and set the absorbing boundary A to be the line $r = b$, $\theta_{\min} \le \theta \le \theta_{\max}$. Unfortunately, given the complicated form of the energy landscape, Eq. (S7) does not easily lend itself to an analytical solution for this choice of R and A. We will work around this problem by describing the escape dynamics from smaller portions of the parameter space, where Eq. (S7) is more amenable to approximation, and then piece together the various results to get a good estimate of the mean bond lifetime. This same approximate piece-wise approach will also yield the survival probability.

B. Partitioning the parameter space into conformational regions

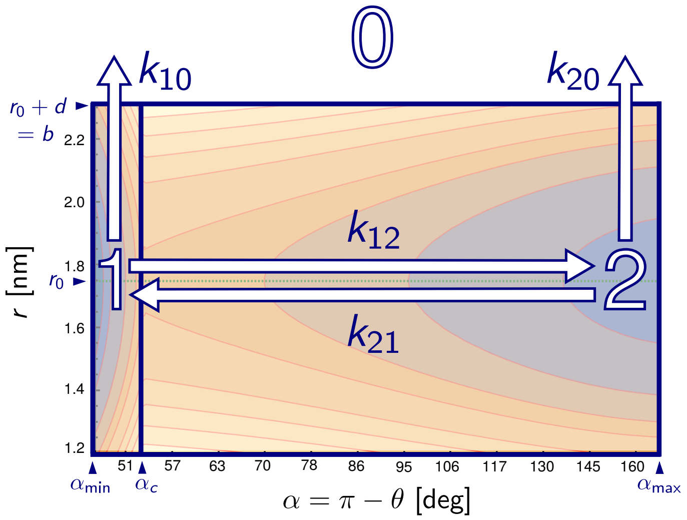

FIG. S1. The energy landscape from Fig. 2 of the main text, partioned into regions of the (r,α) parameter space that reflect different conformational states: state 0 corresponding to the bond ruptured, state 1 corresponding to the bond intact with angle $\alpha_{\min} \le \alpha < \alpha_c$, and state 2 corresponding to the bond intact with angle $\alpha_c \le \alpha \le \alpha_{\max}$. The arrows depict the transition rates k_{10}, k_{20}, k_{12}, and k_{21} between the various states, described in the text.

The energy landscape of Eqs. (S1)-(S2) allows us to partition the (r,θ) parameter space, or equivalently the space of $(r, \alpha = \pi - \theta)$, into domains representing different conformational states, as illustrated in Fig. S1. The region where the bond is intact $(r < b)$ and the angle α is small $(\alpha_{\min} \le \alpha < \alpha_c)$ is denoted as state 1, the corresponding region with an intact bond and large angle $(\alpha_c \le \alpha \le \alpha_{\max})$ is denoted as state 2, and the region where the bond is

ruptured ($r \geq b$) as state 0. If the bond is intact at time $t = 0$, the dynamics of the system will consist of diffusion on the energy landscape, possibly making a number of transitions between states 1 and 2, before eventually the $r = b$ boundary is crossed and bond rupture occurs upon entry to state 0.

The diffusive dynamics exhibit a separation of time scales: the time to equilibrate within each state is typically $< r_0^2/D \sim 20$ ns for the biologically relevant parameter ranges we consider, which is orders of magnitude smaller than the typical times to escape each state (which can be as large as ~ 1 s for the energy barriers in our case). We can thus assume that upon entering either state 1 or state 2, the system will rapidly be driven toward the bottom of the corresponding energy well, and spend considerable amounts of time in the vicinity of the local energy minimum, with brief excursions up the slopes of the well (eventually one of which will carry it into the bond rupture region, or a transition to the other angular state).

Given this partitioning of the parameter space, we can define escape rates from the different states through different boundaries, related to the reciprocal of the escape MFPT introduced in the previous section. These rates (k_{10}, k_{20}, k_{12}, and k_{21}) are depicted as transition arrows in Fig. S1. Let k_{10} be the probability per unit time to escape state 1 to state 0 through the bond rupture ($r = b$) boundary, conditioned on not passing through state 2. We set $k_{10} = 1/\tau_{10}(r_1, \theta_1)$, where τ_{10} is the solution to Eq. (S7) with the region R corresponding to state 1, an absorbing boundary A at the border with state 0 ($r = b$) and a reflecting boundary condition imposed at the border with state 2 ($\alpha = \alpha_c$). The starting position (r_1, θ_1) is the position of the local minimum of the effective potential $V(r, \theta)$ in state 1. Using any other starting position in the vicinity will not substantially alter the result, given the fast equilibration time in the well. We define the escape rate from state 2 to state 0, conditioned on not passing through state 1, as $k_{20} = 1/\tau_{20}(r_2, \theta_2)$, with R being state 2, an absorbing boundary A at the border to state 0, and a reflecting boundary at the border to state 1. The position (r_2, θ_2) corresponds to the local minimum of $V(r, \theta)$ in state 2. The interwell transition rates k_{12} and k_{21} are defined analogously: $k_{12} = 1/\tau_{12}(r_1, \theta_1)$ is the escape rate from state 1 to state 2, conditioned on the bond not rupturing (reflecting boundary at $r = b$), and $k_{21} = 1/\tau_{21}(r_2, \theta_2)$ is the reverse rate from state 2 to state 1.

In the next two sections (1.3 and 1.4) we will find approximate expressions for k_{10}, k_{20}, k_{12} and k_{21} from Eq. (S7), and then in section 1.5 we will show how we can put these together to get analytical results for the mean bond lifetime $\tau(F)$ and survival probability $\Sigma_F(t)$.

C. Deriving expressions for k_{10} and k_{20}

To find an expression for $k_{10} = 1/\tau_{10}(r_1, \theta_1)$, we note that τ_{10} satisfies Eq. (S7) with the region R corresponding to $r < b$, $\alpha_{\min} \leq \alpha < \alpha_c$. The α angular range is equivalent to $\theta_c < \theta \leq \theta_{\max}$. We impose reflecting boundary conditions at θ_c and $\theta_{\max}$, and there is a natural reflecting boundary condition at $r = 0$ because of the logarithmic term in the definition of $V(r, \theta)$ in Eq. (S6). The absorbing boundary A is $r = b$, the border with state 0. We will reduce the dimensionality of the problem by integrating both sides of Eq. S7 over the θ range of R,

$$D \frac{\partial}{\partial r} \int_{\theta_c}^{\theta_{\max}} d\theta \, e^{-V(r,\theta)} \frac{\partial}{\partial r} \tau_{10}(r, \theta) = -\int_{\theta_c}^{\theta_{\max}} d\theta \, e^{-V(r,\theta)}. \tag{S8}$$

The second term on the left hand side in Eq. S7 vanishes after the integration because $\exp(-V(r, \theta)) = 0$ at $\theta = \theta_c$ and $\theta = \theta_{\max}$ because of the reflecting boundary conditions.

Because of the $e^{-V(r,\theta)}$ terms inside the integrals on both sides of Eq. (S8), the dominant contribution to the integrals at any particular value of r occurs when $V(r, \theta)$ reaches a minimum with respect to θ inside the state 1 range $\theta_c < \theta \leq \theta_{\max}$. This happens at some value of $\theta = \theta_{m1}(r)$ for a given r. Thus we can treat Eq. (S8) as an equation for $\tau_{10}(r, \theta_{m1}(r))$, which we will write in condensed notation as just $\tau_{10}(r)$. Note that we are interested in $\tau_{10}(r_1)$, since $\theta_1 = \theta_{m1}(r_1)$ is just the position of the well minimum at r_1. In this approximation Eq. (S8) becomes

$$D \frac{\partial}{\partial r} \left[e^{-\widetilde{V}_1(r)} \frac{\partial}{\partial r} \tau_{10}(r) \right] = -e^{-\widetilde{V}_1(r)}, \tag{S9}$$

where the effective 1D potential $\widetilde{V}_1(r)$ is given by

$$\widetilde{V}_1(r) = -\ln \left[\int_{\theta_c}^{\theta_{\max}} d\theta e^{-V(r,\theta)} \right]. \tag{S10}$$

With the absorbing boundary condition $\tau_{10}(b) = 0$, Eq. S9 can be solved for $\tau_{10}(r)$,

$$\tau_{10}(r) = \frac{1}{D} \int_r^b dr' \, e^{\widetilde{V}_1(r')} \int_0^{r'} dr'' e^{-\widetilde{V}_1(r'')}. \tag{S11}$$

The function $\widetilde{V}_1(r')$ is a monotonically increasing function of r' at large r'. Due to the presence of the $\exp(\widetilde{V}_1(r'))$ term, the integral over r' in Eq. S11 gets its dominant contribution from r' near the upper limit b. Conversely because of the $\exp(-\widetilde{V}_1(r''))$ term, the integral over r'' gets its dominant contribution near $\tilde{r}_1$, the position where $\widetilde{V}_1(r)$ reaches a minimum. To simplify Eq. (S11), we thus make two approximations: (i) expand $\widetilde{V}_1(r') \approx \widetilde{V}_1(b) + \widetilde{V}_1'(b)(r'-b)$; (ii) assume $b \gg \tilde{r}_1$, so the upper limit in the integral over r'' can be replaced by ∞. Along similar lines, if the starting position is $r = r_1$, the precise value of the lower limit on the r' integral has a negligible effect on the result, so we can replace it with 0. Eq. (S11) can then be evaluated to yield an expression for $\tau_{10}(r_1)$,

$$\tau_{10}(r_1) \approx \frac{e^{\widetilde{V}_1(b)}}{D\widetilde{V}_1'(b)} \int_0^\infty dr''\, e^{-\widetilde{V}_1(r'')}, \tag{S12}$$

where we have kept the largest contributions to the result. The right-hand side does not have a dependence on the value of the starting position r_1, consistent with the assumption of fast equilibration within the well. We denote the integral in Eq. S12 as

$$\widetilde{Z}_1 \equiv \int_0^\infty dr'' e^{-\widetilde{V}_1(r'')}, \tag{S13}$$

which needs to be evaluated to get a closed form expression for $\tau_{10}(r_1)$. Because this cannot be done exactly, we will use a saddle-point approximation by expanding $\widetilde{V}_1(r)$ around its minimum at $r = \tilde{r}_1$,

$$\widetilde{Z}_1 \approx e^{-\widetilde{V}_1(\tilde{r}_1)} \sqrt{\frac{2\pi}{\widetilde{V}_1''(\tilde{r}_1)}} \approx e^{-\widetilde{V}_1(\tilde{r}_1)} \sqrt{\frac{2\pi}{k_0}}, \tag{S14}$$

where we have used the fact that $\widetilde{V}_1''(\tilde{r}_1) \approx k_0$ in the limit of large k_0 (which is valid when the barrier to rupture $E_0 \gg k_B T$, since $E_0 = (k_0 + k_1(1+\cos\theta_{\max}))d^2/2$).

Putting everything together from Eqs. (S12)-(S14), we can find $\tau_{10}(r_1)$ explicitly. This requires carrying out $\widetilde{V}_1$ integrals using the definition of Eq. (S10), and approximating $\tilde{r}_1 \approx r_0$. This leads to a final expression for k_{10}:

$$k_{10} = \frac{1}{\tau_{10}(r_1)} = \frac{\beta_1}{\alpha_1}, \tag{S15}$$

where

$$\begin{aligned} \alpha_1 &\equiv \sqrt{\pi}(dr_0)^2(\Delta_1(2bF - \tilde{E}_1) - 2H)\left(e^{\frac{cFr_0(c-2\mu)+2H\mu}{\Delta_1}} - e^{\frac{cH+\mu(H-Fr_0\mu)}{\Delta_1}}\right) e^{\frac{c(-2F(bc+d\mu)+\tilde{E}_1\Delta_3+2\tilde{E}_0+\tilde{E}_1)}{2\Delta_1}}, \\ \beta_1 &\equiv 4b^2 D\tilde{E}_0^{3/2}\,(H - Fr_0\Delta_1)\left(e^{\frac{c^2(\tilde{E}_1-2bF)+2H\Delta_3+\mu(\mu(\tilde{E}_1-2bF)+2\tilde{E}_0+\tilde{E}_1)}{2\Delta_1}} - e^{\frac{\mu(2c(\tilde{E}_1-2bF)+4H+2\tilde{E}_0+\tilde{E}_1)}{2\Delta_1}}\right). \end{aligned} \tag{S16}$$

In the above expressions, as well as the ones for the other rates below, we will use a set of abbreviated notations, as follows:

$$\begin{aligned} &c \equiv \cos\theta_c, \quad \mu \equiv \cos\theta_{\max}, \quad \nu \equiv \cos\theta_{\min}, \\ &\Delta_1 \equiv c-\mu, \quad \Delta_2 \equiv c-\nu, \quad \Delta_3 \equiv c+\mu, \quad \Delta_4 \equiv \mu+\nu, \\ &\tilde{E}_0 \equiv E_0 - \frac{1+\mu}{\nu-\mu}E_1, \quad \tilde{E}_1 \equiv \frac{2E_1}{\nu-\mu}. \end{aligned} \tag{S17}$$

For $k_{20} = 1/\tau_{20}(r_2,\theta_2)$, the derivation proceeds exactly analogously to the one for k_{10}, except that the region R now corresponds to $r < b$, $\theta_{\min} \le \theta \le \theta_c$. The final expression for k_{20} is:

$$k_{20} = \frac{1}{\tau_{20}(r_2)} = \frac{\beta_2}{\alpha_2}, \tag{S18}$$

where

$$\begin{aligned} \alpha_2 &\equiv dr_0^2\sqrt{\frac{\pi}{\tilde{E}_0}}(\Delta_2(\tilde{E}_1 - 2bF) - 2(G-H))^2\left(1 - e^{Fr_0\Delta_2+G-H}\right) e^{\frac{c(\nu(\tilde{E}_1-2dF)+E_2)-\nu(2Fr_0\nu+E_2-2(G-H))}{2\Delta_2}}, \\ \beta_2 &\equiv 4b^2 D\tilde{E}_0^{3/2}\,(Fr_0\Delta_2 + G - H)\left(e^{\frac{\nu(\nu(\tilde{E}_1-2bF)+2(G-H))}{2\Delta_2}} - e^{-\frac{c((\tilde{E}_1-2bF)(c-2\nu)-2(G-H))}{2\Delta_2}}\right). \end{aligned} \tag{S19}$$

D. Deriving expressions for k_{12} and k_{21}

Let us first consider the transition rate $k_{12} = 1/\tau_{12}(r_1, \theta_1)$ from state 1 to state 2, conditioned on the bond not rupturing. The starting point (r_1, θ_1) is at the local energy minimum in state 1, and we will place the absorbing boundary at some angle $\theta < \theta_c$, beyond the angular energy barrier at θ_c that defines the border with state 2. Once we are not in the immediate vicinity of the barrier top, the precise location of the absorbing boundary within state 2 does not significantly change the value of τ_{12}. This is because once the system has overcome the barrier to transition from state 1 to state 2, it rapidly descends into the state 2 energy well. Using this freedom, we will choose the absorbing boundary A at $\theta = \theta_2$, the position of the local energy minimum in the state 2 well.

We choose the region R to have an r range between 0 and b, with a reflecting boundary imposed at $r = b$. The logarithmic term in $V(r, \theta)$ in Eq. (S6) provides another reflecting boundary at $r = 0$. Integrating both sides of Eq. S7 over this r range gives:

$$D\frac{\partial}{\partial\theta}\int_0^b dr\,\frac{1}{r^2}e^{-V(r,\theta)}\frac{\partial}{\partial\theta}\tau_{12}(r,\theta) = -\int_0^b dr\,e^{-V(r,\theta)}. \tag{S20}$$

The first term in Eq. S7 vanishes under integration because of the reflecting boundary conditions.

The dominant contribution to the integral on the left-hand side of Eq. S20 for a given angle θ occurs at $r = r_m(\theta)$, where $V(r, \theta)$ is minimal with respect to r at that θ. To a good approximation $r_m(\theta) \approx r_0$ for the force and parameter ranges we consider. We can thus treat Eq. (S20) as an effective equation for $\tau_{12}(r_0, \theta)$, which we will denote compactly as $\tau_{12}(\theta)$. We are ultimately interested in getting an expression for $k_{12} = 1/\tau_{12}(\theta_1)$. Using this approximation, we can rewrite Eq. (S20) as:

$$D_\theta\frac{\partial}{\partial\theta}\left[e^{-\widetilde{V}(\theta)}\frac{\partial}{\partial\theta}\tau_{12}(\theta)\right] = -e^{-\widetilde{V}(\theta)}, \tag{S21}$$

where $D_\theta \equiv D/r_0^2$, and the effective 1D potential $\widetilde{V}(\theta)$ is given by

$$\widetilde{V}(\theta) = -\ln\left[\int_0^b dr\,e^{-V(r,\theta)}\right] \approx V(r_0,\theta) \tag{S22}$$

In the second expression on the right, we have kept only the most significant contribution from the saddle-point approximation of the integral.

With the absorbing boundary condition $\tau_{12}(\theta_2) = 0$, Eq. S21 can be solved for $\tau_{12}(\theta)$:

$$\tau_{12}(\theta) = \frac{1}{D_\theta}\int_{\theta_2}^{\theta} d\theta'\,e^{\widetilde{V}(\theta')}\int_{\theta'}^{\theta_{\max}} d\theta''\,e^{-\widetilde{V}(\theta'')}. \tag{S23}$$

Substituting $\widetilde{V}(\theta) \approx V(r_0, \theta) = U(r_0, \theta) - k_BT\ln(r_0^2 \sin\theta)$ allows us to rewrite Eq. (S23) in terms of integration variables $\cos\theta'$ and $\cos\theta''$. The MFPT $\tau_{12}(\theta_1)$ from starting position θ_1 is then

$$\begin{aligned}\tau_{12}(\theta_1) &= \frac{1}{D_\theta}\int_{\cos\theta_1}^{\cos\theta_2} d(\cos\theta')\,e^{U(r_0,\theta')-\ln\sin^2\theta'}\int_{\mu}^{\cos\theta'} d(\cos\theta'')\,e^{-U(r_0,\theta'')},\\ &\approx \frac{1}{D_\theta}\int_{\cos\theta_1}^{\cos\theta_2} d(\cos\theta')\,e^{U(r_0,\theta')}\int_{\mu}^{\cos\theta'} d(\cos\theta'')\,e^{-U(r_0,\theta'')},\\ &\equiv \frac{1}{D_\theta}\int_{\cos\theta_1}^{\cos\theta_2} d(\cos\theta')\,e^{U(r_0,\theta')}Z_{12}(\cos\theta').\end{aligned} \tag{S24}$$

In the second line we have neglected the $-\ln\sin^2\theta$ contribution in the first exponential, since it does not significantly change the value of $\tau_{12}(\theta_1)$, and in the third line we have introduced the function $Z_{12}(\cos\theta') \equiv \int_{\mu}^{\cos\theta'} d(\cos\theta'')\,e^{-U(r_0,\theta'')}$. The final approximation is to note that θ_1 is close to $\theta_{\max}$, and θ_2 is close to $\theta_{\min}$, so we can replace $\cos\theta_1$ in the integration bounds with $\mu = \cos\theta_{\max}$, and replace $\cos\theta_2$ with $\nu = \cos\theta_{\min}$. Thus the final integral for $\tau_{12}(\theta_1)$ takes the form,

$$\tau_{12}(\theta_1) \approx \frac{1}{D_\theta}\int_{\mu}^{\nu} d(\cos\theta')\,e^{U(r_0,\theta')}Z_{12}(\cos\theta'). \tag{S25}$$

Since the angular dependence of $U(r_0,\theta')$ from Eqs. (S1)-(S2) is explicitly in terms of $\cos\theta'$, the integration variable, it turns out the integral in Eq. (S25) can be evaluated exactly, to yield a rather complex (but closed form) expression for k_{12},

$$k_{12} = \frac{1}{\tau_{12}(\theta_1)} = \frac{D(H - Fr_0\Delta_1)^2}{r_0^2\left(\alpha_3^a\left(\beta_3^a + \beta_3^b + \beta_3^c + \beta_3^d\right) + \alpha_3^b\left(\gamma_3^a + \gamma_3^b + \gamma_3^c\right)\right)}, \tag{S26}$$

where,

$$\begin{aligned}
\alpha_3^a &\equiv \frac{\Delta_2\left(Fr_0\Delta_1 - H\right)e^{-\frac{cH}{\Delta_1} - \frac{\nu(Fr_0\Delta_2+G)}{\Delta_2}}}{\left(Fr_0\Delta_2 + G - H\right)^2} + \Delta_1^2\left(e^{H - Fr_0\Delta_1} + Fr_0\Delta_1 - H - 1\right),\\
\alpha_3^b &\equiv \frac{\Delta_2\left(H - Fr_0\Delta_1\right)e^{-\frac{cFr_0\Delta_2 + c(G-H)+H\nu}{\Delta_2} - \frac{cH}{\Delta_1}}}{\left(Fr_0\Delta_2 + G - H\right)^2},\\
\beta_3^a &\equiv c^2 Fr_0\left(Fr_0\nu e^{\frac{cH}{\Delta_1} + Fr_0\nu + \frac{G\nu}{\Delta_2}} + e^{\frac{cG}{\Delta_2} + \frac{cH}{\Delta_1} + Fr_0\mu} - e^{cFr_0 + \frac{cG}{\Delta_2} + \frac{H\mu}{\Delta_1}} + e^{\frac{cFr_0\Delta_2 + c(G-H)+H\nu}{\Delta_2} + \frac{cH}{\Delta_1}}\right),\\
\beta_3^b &\equiv H\nu e^{\frac{cH}{\Delta_1} + \frac{H\nu}{\Delta_2}}\left(e^{cFr_0 + \frac{c(G-H)}{\Delta_2}} + \left(Fr_0\nu - G + H\right)e^{\frac{\nu(Fr_0\Delta_2 + G - H)}{\Delta_2}}\right),\\
\beta_3^c &\equiv \mu\left(\left(G - H - Fr_0\nu\right)e^{cFr_0 + \frac{cG}{\Delta_2} + \frac{H\mu}{\Delta_1}} + \left(Fr_0\nu - G + H\right)e^{\frac{cG}{\Delta_2} + \frac{cH}{\Delta_1} + Fr_0\mu}\right.\\
&\qquad\left. + Fr_0\nu\left(Fr_0\nu - G + H\right)e^{\frac{cH}{\Delta_1} + Fr_0\nu + \frac{G\nu}{\Delta_2}} + Fr_0\nu e^{\frac{cFr_0\Delta_2 + c(G-H)+H\nu}{\Delta_2} + \frac{cH}{\Delta_1}}\right),\\
\beta_3^d &\equiv -c\left(Fr_0\nu\left(Fr_0\Delta_4 - G + 2H\right)e^{\frac{cH}{\Delta_1} + Fr_0\nu + \frac{G\nu}{\Delta_2}} + \left(G - H - Fr_0\Delta_4\right)e^{cFr_0 + \frac{cG}{\Delta_2} + \frac{H\mu}{\Delta_1}}\right.\\
&\qquad\left. + \left(Fr_0\Delta_4 - G + H\right)e^{\frac{cG}{\Delta_2} + \frac{cH}{\Delta_1} + Fr_0\mu} + \left(Fr_0\Delta_4 + H\right)e^{\frac{cFr_0\Delta_2 + c(G-H)+H\nu}{\Delta_2} + \frac{cH}{\Delta_1}}\right),\\
\gamma_3^a &\equiv e^{\frac{c(G-H)}{\Delta_2}}\left(c^2Fr_0\left(\left(cFr_0 + 1\right)e^{cFr_0 + \frac{cH}{\Delta_1} + \frac{H\nu}{\Delta_2}} + e^{\frac{cH}{\Delta_2} + \frac{cH}{\Delta_1} + Fr_0\mu} - e^{cFr_0 + \frac{cH}{\Delta_2} + \frac{H\mu}{\Delta_1}}\right)\right.\\
&\qquad\left. + H\left(cFr_0\nu - c(G-H) + c_{\max}\right)e^{cFr_0 + \frac{cH}{\Delta_1} + \frac{H\nu}{\Delta_2}}\right),\\
\gamma_3^b &\equiv \mu e^{\frac{cG}{\Delta_2}}\left(\left(G - H - Fr_0\nu\right)\left(e^{cFr_0 + \frac{H\mu}{\Delta_1}} - e^{\frac{cH}{\Delta_1} + Fr_0\mu}\right) + Fr_0\left(cFr_0\nu - c(G-H) + \nu\right)e^{\frac{cFr_0\Delta_2 - cH + H\nu}{\Delta_2} + \frac{cH}{\Delta_1}}\right),\\
\gamma_3^c &\equiv -ce^{\frac{c(G-H)}{\Delta_2}}\left(\left(cF^2r_0^2\Delta_4 + Fr_0(-cG + 2cH + \Delta_4) + H\right)e^{cFr_0 + \frac{cH}{\Delta_1} + \frac{H\nu}{\Delta_2}}\right.\\
&\qquad\left. + \left(G - H - Fr_0\Delta_4\right)e^{cFr_0 + \frac{cH}{\Delta_2} + \frac{H\mu}{\Delta_1}} + \left(Fr_0\Delta_4 - G + H\right)e^{\frac{cH}{\Delta_2} + \frac{cH}{\Delta_1} + Fr_0\mu}\right).
\end{aligned} \tag{S27}$$

To get the transition rate k_{21} from state 2 back to 1, we note that a physically consistent model should relate k_{12} and k_{21} to each other through detailed balance. The quasi-equilibrium probability ratio of being in state 1 relative to state 2 (in the long-time limit, conditioned on the bond not rupturing), is approximately $Z_{12}(c)/Z_{21}(c)$, where $c = \cos\theta_c$ and $Z_{21}(\cos\theta') \equiv \int_{\cos\theta'}^{\nu} d(\cos\theta'')\, e^{-U(r_0,\theta'')}$. Thus we can write:

$$k_{21} = k_{12}\frac{Z_{12}(c)}{Z_{21}(c)} = k_{12}\frac{\Delta_1\left(e^{Fr_0\Delta_1 - H} - 1\right)\left(Fr_0\Delta_2 + G - H\right)e^{\frac{cG + Fr_0\mu\Delta_2 - H\nu}{\Delta_2}}}{\Delta_2\left(Fr_0\Delta_1 - H\right)\left(e^{\frac{\nu(Fr_0\Delta_2 + G - H)}{\Delta_2}} - e^{\frac{c(Fr_0\Delta_2 + G - H)}{\Delta_2}}\right)}. \tag{S28}$$

Eq. (S28), together with the expressions in Eqs. (S26)-(S27), gives a complete closed form result for k_{21}.

E. Survival probability and mean bond lifetime

The final part of the derivation involves expressing the survival probability $\Sigma_F(t)$ and mean bond lifetime $\tau(F)$ in terms of the four rates k_{10}, k_{20}, k_{12}, and k_{21}, following a standard approach to first passage problems in discrete state kinetic networks [1]. As mentioned earlier, all these four rates are themselves functions of F, as can be seen in the results of the previous two sections, but for simplicity we do not show the F dependence explicitly.

Consider the probability $S_i(t)$ that the bond survived intact until time t, given that the system started in state $i = 1, 2$ at time $t = 0$. If we discretize time in infinitesimal steps of δt, with $t = n\delta t$, then the probability $S_1(t)$ can

be written as

$$S_1(n\delta t) = [1-(k_{10}+k_{12})\delta t]^n + \sum_{m=0}^{n} [1-(k_{10}+k_{12})\delta t]^m \, k_{12}\delta t \, S_2((n-m)\delta t). \tag{S29}$$

The right-hand side of Eq. (S29) can be understood as follows: $k_{10}\delta t$ is the probability to transition from 1 to 0 in time step δt, and $k_{12}\delta t$ is the probability to transition from 1 to 2 in time step δt. Thus we see that the first term on the right-hand side of Eq. (S29) is the probability that the bond survived without either rupturing or transitioning to state 2 for the entire n time steps. This is one contribution to $S_1(n\delta t)$. However there is another contribution, since the bond could still survive, but make at least one transition to state 2 during those n steps. The sum in Eq. (S29) is this second contribution, consisting of the cases where the bond does not leave state 1 for m time steps, then makes a transition to state 2, and survives the remaining $n-m$ time steps. The last probability is just $S_2((n-m)\delta t)$. Taking the limit $\delta t \to 0$, $n = t/\delta t \to \infty$, we can rewrite Eq. (S29) as

$$S_1(t) = e^{-(k_{10}+k_{12})t} + \int_0^t dt' e^{-(k_{10}+k_{12})t'} k_{12} S_2(t-t'). \tag{S30}$$

An exactly analogous argument for $S_2(t)$ yields a second integral equation,

$$S_2(t) = e^{-(k_{20}+k_{21})t} + \int_0^t dt' e^{-(k_{20}+k_{21})t'} k_{21} S_1(t-t'). \tag{S31}$$

The system of equations, Eq. (S30)-(S31), can be solved by first applying a Laplace transform, $\tilde{S}_i(s) \equiv \int_0^\infty dt\, e^{-st} S_i(t)$. This gives

$$\begin{aligned} \tilde{S}_1(s) &= \frac{1}{k_{10}+k_{12}+s} + \frac{k_{12}\tilde{S}_2(s)}{k_{10}+k_{12}+s}, \\ \tilde{S}_2(s) &= \frac{1}{k_{20}+k_{21}+s} + \frac{k_{21}\tilde{S}_1(s)}{k_{20}+k_{21}+s}. \end{aligned} \tag{S32}$$

The solutions for $\tilde{S}_1(s)$ and $\tilde{S}_2(s)$ are then:

$$\begin{aligned} \tilde{S}_1(s) &= \frac{k_{12}+k_{20}+k_{21}+s}{k_{10}k_{20}+k_{12}k_{20}+k_{21}k_{10}+(k_{10}+k_{20}+k_{12}+k_{21})s+s^2}, \\ \tilde{S}_2(s) &= \frac{k_{21}+k_{10}+k_{12}+s}{k_{10}k_{20}+k_{12}k_{20}+k_{21}k_{10}+(k_{10}+k_{20}+k_{12}+k_{21})s+s^2}. \end{aligned} \tag{S33}$$

Before going further, note that if the system started in state i at time $t=0$, the probability to rupture between times t and $t+\delta t$ is just $S_i(t) - S_i(t+\delta t) \approx -\delta t\, dS_i(t)/dt$. Hence the mean time to rupture τ_i given starting state i is just

$$\tau_i = -\int_0^\infty dt\, t \frac{dS_i}{dt} = \int_0^\infty dt\, S_i(t) = \tilde{S}_i(0). \tag{S34}$$

The second equality follows from integration by parts, and the fact that $S_i(0) = 1$, $S_i(\infty) = 0$. Plugging $s = 0$ into Eq. (S33) thus gives

$$\begin{aligned} \tau_1 &= \frac{k_{12}+k_{20}+k_{21}}{k_{10}k_{20}+k_{12}k_{20}+k_{21}k_{10}}, \\ \tau_2 &= \frac{k_{21}+k_{10}+k_{12}}{k_{10}k_{20}+k_{12}k_{20}+k_{21}k_{10}}. \end{aligned} \tag{S35}$$

To get the final expression for the mean bond lifetime τ, we need the initial probabilities p_i^0 of being in state i at time $t=0$. Since we assume the system has quasi-equilibrated at $F=0$ before the application of force at $t>0$, the ratio p_1^0/p_2^0 is just $Z_{12}(c)/Z_{21}(c)$ evaluated at $F=0$. From this we get the following probabilities:

$$p_1^0 = \frac{\Delta_1\left(e^H-1\right)(G-H)e^{\frac{cG}{\Delta_2}}}{\Delta_1\left(e^H-1\right)(G-H)e^{\frac{cG}{\Delta_2}} + \Delta_2 H\left(e^{\frac{G\nu}{\Delta_2}+H} - e^{\frac{c(G-H)+H(\Delta_2+\nu)}{\Delta_2}}\right)}, \qquad p_2^0 = 1-p_1^0. \tag{S36}$$

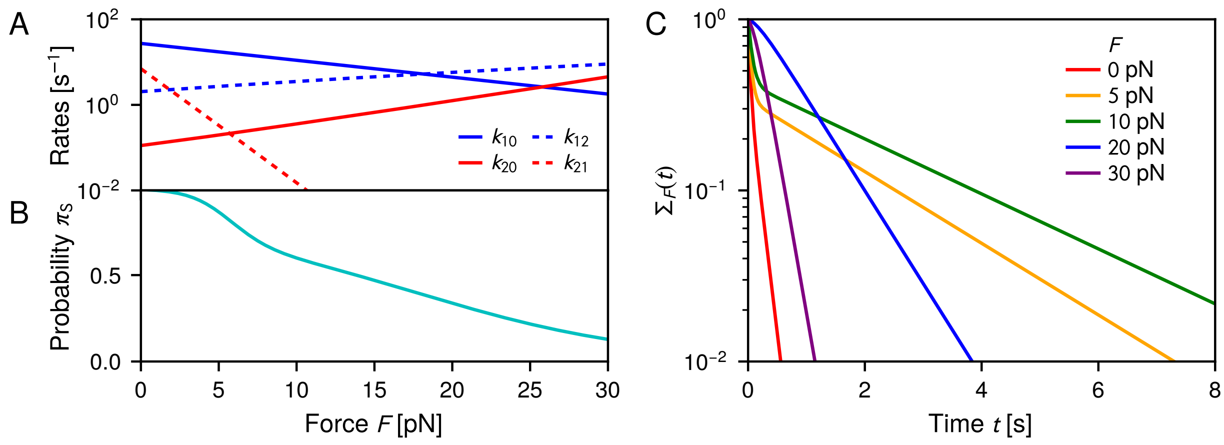

FIG. S2. A: The transition rates k_{10}, k_{20} (solid lines, Eqs. (S15)-(S19)) and k_{12}, k_{21} (dashed lines, Eqs. (S26)-(S28)) as a function of force F. B: The probability π_S (Eqs. (S41)-(S42)) to be in state 1 (small α) at rupture versus F. C: The survival probability $\Sigma_F(t)$, Eqs. (S38)-(S39), as a function of time t, for selected forces F indicated in the legend. For all panels the results are plotted for the best-fit parameters given in Table 1 of the main text.

For the best-fit parameters given in Table 1 of the main text, these probabilities are $p_1^0 = 0.77$, $p_2^0 = 0.23$. The initial small α probability was denoted as $p_S^0 \equiv p_1^0$ in the main text. The mean bond lifetime, weighting over all possible starting states, is given by

$$\tau = p_1^0 \tau_1 + p_2^0 \tau_2. \tag{S37}$$

Eq. (S37), supplemented by Eqs. (S35)-(S36) and the expressions for k_{10}, k_{20}, k_{12}, and k_{21} from the previous two sections (Eqs. (S15)-(S19), (S26)-(S28)), constitutes the complete theoretical result for τ.

Similarly the survival probability $\Sigma_F(t)$ is just the weighted sum of $S_1(t)$ and $S_2(t)$,

$$\Sigma_F(t) = p_1^0 S_1(t) + p_2^0 S_2(t), \tag{S38}$$

where $S_i(t)$ can be found by inverse Laplace transforming the solutions from Eq. (S33),

$$S_i(t) = e^{-\sigma t/2} \left[\cosh\left(\frac{t}{2}\sqrt{\sigma^2 - 4\rho}\right) + \frac{2\lambda_i - \sigma}{\sqrt{\sigma^2 - 4\rho}} \sinh\left(\frac{t}{2}\sqrt{\sigma^2 - 4\rho}\right) \right]. \tag{S39}$$

Here $\rho \equiv k_{10}k_{20} + k_{12}k_{20} + k_{10}k_{21}$, $\sigma \equiv k_{10} + k_{20} + k_{12} + k_{21}$, $\lambda_1 \equiv \sigma - k_{10}$, $\lambda_2 \equiv \sigma - k_{20}$. Note that because the cosh and sinh share the same argument, Eq. (S39) can also be expressed in terms of two distinct exponential contributions with different prefactors. This is what leads to the double-exponential behavior seen in the survival probabilities in the main text.

Another aspect mentioned in the main text is the final conformational state of the system at the moment of rupture, whether it is state 1 (small α) or state 2 (large α). If the system could quasi-equilibrate at the applied force F before rupture occurred (i.e. if the rupture rates k_{10} and k_{20} were sufficiently small), the probability of being in state 1 at rupture would be $p_S = k_{21}/(k_{12} + k_{21})$. For the case $F = 15.1$ pN, discussed in the main text, $p_S = 10^{-4}$ for the parameter values of Table 1. In reality, however, the system does not have time to fully quasi-equilibrate, and the actual probability of being in state 1 at rupture is 0.47 for this particular value of F. To derive this number, we define the splitting probability π_{i1}, the probability that the system will rupture in state 1, given a starting state i at time $t = 0$. The splitting probabilities π_{11} and π_{21} satisfy the identities [1]:

$$\pi_{11} = \frac{k_{10}}{k_{10} + k_{12}} + \frac{k_{12}}{k_{10} + k_{12}}\pi_{21}, \qquad \pi_{21} = \frac{k_{21}}{k_{20} + k_{21}}\pi_{11}. \tag{S40}$$

The first identity states the π_{11} involves two contributions: starting in state 1, the system can either rupture before jumping to state 2 (probability $\frac{k_{10}}{k_{10}+k_{12}}$) or jump to state 2 first (probability $\frac{k_{12}}{k_{10}+k_{12}}$) and then eventually make it back to state 1 to rupture (probability π_{21}). Similarly for the second identity, π_{21} is equal to the probability of jumping to state 1 before rupture ($\frac{k_{21}}{k_{20}+k_{21}}$) times π_{11}. Eq. (S40) can be solved for π_{11} and π_{21},

$$\pi_{11} = \frac{k_{10}(k_{20} + k_{21})}{\rho}, \qquad \pi_{21} = \frac{k_{10}k_{21}}{\rho}. \tag{S41}$$

The probability of rupturing from state 1 independent of initial state (denoted as $\pi_{\rm S}$ in the main text) is

$$\pi_{\rm S} = \pi_{11} p_1^0 + \pi_{21} p_2^0 \tag{S42}$$

For $F = 15.1$ pN we get $\pi_{\rm S} = 0.47$.

Fig. S2 illustrates some of the physical quantities described up to now. Panel A shows the transition rates k_{10}, k_{20}, k_{12}, and k_{21} versus F for the best-fit parameters of Table 1 in the main text. Panel B shows the corresponding probabilties $\pi_{\rm S}$ and panel C depicts the survival probabilities $\Sigma_F(t)$ for various F. At $F = 0$, the rupture is almost entirely from state 1, with $\pi_{\rm S} \approx 1$. Hence $\Sigma_F(t)$ at $F = 0$ is dominated by a single exponential contribution, $\Sigma_F(t) \approx \exp(-k_{10}(F)t)$ (red curve in Fig. S2C). The probability $\pi_{\rm S}$ decreases with F, and in the opposite extreme of large F, the rupture occurs mainly from state 2. In this regime we again have approximately single exponential survival probabilities, $\Sigma_F(t) \approx \exp(-k_{20}(F)t)$, as seen for example in Fig. S2C for 30 pN (purple curve). At intermediate forces, where the chances of rupture from either state are comparable, we have double-exponential behavior in $\Sigma_F(t)$, as seen for $F = 5$, 10 pN (orange and green curves in Fig. S2C). The latter cases distinctly show two different linear slopes in the logarithmic plot of Fig. S2C, in contrast to the small and large force regimes, where the decay of $\ln \Sigma_F(t)$ is dominated by a single linear slope.

The final quantity discussed in the main text is τ_L, the mean duration of the large α conformation (state 2), measured from the first entrance into the state until either rupture occurs or the system transitions to state 1. Since the total escape rate from state 2 is $k_{20} + k_{21}$, the probability of leaving state 2 between times t and $t + \delta t$, where $t = 0$ is the time of entrance, is: $\delta t(k_{20} + k_{21}) \exp(-(k_{20} + k_{21})t)$. Thus the mean duration is:

$$\tau_L = \int_0^\infty dt\, t(k_{20} + k_{21}) e^{-(k_{20}+k_{21})t} = \frac{1}{k_{20} + k_{21}}. \tag{S43}$$

II. CONSISTENCY WITH AN EARLIER CATCH BOND MODEL

One of the nice features of our approach is its generality: even though we focus in the main text on a system with a substantial angular barrier H, the theoretical model continues to hold even in the absence of such a barrier. In this section we show that the $\tau(F)$ expression for an earlier, barrier-less model for catch bonds in selectin systems, introduced and numerically verified in Ref. [2], is just a special case of our more general $\tau(F)$.

For the selectin case, the full angular range was used, so $\theta_{\rm min} = 0°$ and $\theta_{\rm max} = \pi$. There was no angular barrier or energy offset, so $H = G = 0$, and we can take $\theta_c = 90°$, since the border between state 1 and state 2 is arbitrary without a barrier present. In this case from Eq. (S17) we see that $\tilde{E}_0 = E_0$ and $\tilde{E}_1 = E_1$. Plugging all these values into the expressions for the transition rates derived above, we find relatively simple results:

$$\begin{aligned}
k_{10} &= \frac{4DE_0^{3/2} F (r_0 + d)^2 e^{-E_0 - E_1/2 - dF} \left(e^{E_1/2} - e^{F(r_0+d)}\right)}{\sqrt{\pi} r_0 d^2 (e^{Fr_0} - 1)(E_1 - 2F(r_0 + d))}, \\
k_{20} &= \frac{4DE_0^{3/2} F (r_0 + d)^2 e^{-E_0 - E_1} \left(e^{E_1/2} - e^{F(r_0+d)}\right)}{\sqrt{\pi} r_0 d^2 (e^{Fr_0} - 1)(E_1 - 2F(r_0 + d))}, \\
k_{12} &= \frac{DF^2 e^{2Fr_0}}{1 + e^{2Fr_0}(2Fr_0 - 1)}, \\
k_{21} &= \frac{DF^2 e^{Fr_0}}{1 + e^{2Fr_0}(2Fr_0 - 1)}.
\end{aligned} \tag{S44}$$

Without an angular barrier the transitions between angular regions are many orders of magnitude faster than the transitions to rupture, as can be seen from the fact that k_{10} and k_{20} both include a factor of e^{-E_0} in the numerator that is not present in k_{12} and k_{21}. Typically the factor $e^{-E_0} \ll 1$ since E_0 sets the overall energy scale for rupture, and $E_0 \sim 17 - 26$ (units of $k_B T$) for the systems considered in Ref. [2]. Hence we can assume in this case that k_{10}, $k_{20} \ll k_{12}$, k_{21}. This simplifies the expressions for τ_1 and τ_2 in Eq. (S35), so that $\tau_1 \approx \tau_2 \approx (k_{12}+k_{21})/(k_{12}k_{20}+k_{21}k_{10})$. Hence the mean bond lifetime is also the same as τ_1 and τ_2,

$$\tau(F) \approx \frac{k_{12} + k_{21}}{k_{12}k_{20} + k_{21}k_{10}} = \frac{\sqrt{\pi} r_0 (E_1 - 2F(r_0 + d)) e^{E_0 + dF} (e^{2Fr_0} - 1)}{4DE_0^{3/2} F (1 + r_0/d)^2 \left(1 - e^{2F(r_0+d) - E_1}\right)}. \tag{S45}$$

This is in complete agreement with the $\tau(F)$ from Eq. (2) in Ref. [2]. The survival probability in this limit becomes a single exponential, $\Sigma_F(t) \approx \exp(-t/\tau(F))$, with $\tau(F)$ from Eq. (S45).

Our derivation of $\tau(F)$ and $\Sigma_F(t)$ in the previous section also allows us to make a comparison to another catch bond model. By partitioning the parameter space into two angular states, and focusing on four transition rates (k_{10}, k_{20}, k_{12}, k_{21}), our approach on the surface seems analogous to the phenomenological two-state catch bond model [3, 4]. However in this phenomenological model each transition rate k_{ij} is assumed to have a simple Bell-like dependence on the force, $k_{ij} = k^0_{ij} \exp(F x_{ij})$, for coefficients k^0_{ij} and distances x_{ij}. Our general expressions for the transition rates in the previous sections, and even the simplified versions of Eq. (S44) in the barrier-less limit, are quite different from Bell models. This is because these rates are derived from an underlying energy landscape based on a structural model. Our parameters thus directly connect to structural / energetic features of the system, in contrast to the k^0_{ij}, x_{ij} parameters of the phenomenological model.

III. MAXIMUM LIKELIHOOD FITTING TO THE EXPERIMENTAL DATA

The experimental data $\mathcal{D}$ (Ref. [5] Fig. 4A) consists of $N = 803$ points, $\mathcal{D} = \{(t_i, F_i), i = 1, \ldots, N\}$, where t_i is the measured bond lifetime, and F_i is the applied force. Let Λ be the set of free parameters in the model other than F. The probability $\mathcal{P}(t_i|F_i, \Lambda)$ of observing the ith bond lifetime, given force F_i and particular set of parameter values Λ, is:

$$\mathcal{P}(t_i|F_i, \Lambda) = -\delta t \left. \frac{d\Sigma_{F_i}(t_i)}{dt} \right|_{\Lambda}, \tag{S46}$$

where $\Sigma_F(t)$ is the survival probability at force F. Since we have an analytical expression for $\Sigma_F(t)$ from Eqs. (S38)-(S39), we also can get an analytical form for $d\Sigma_F(t)/dt$, which allows us to evaluate $\mathcal{P}(t_i|F_i, \Lambda)$. The joint probability of the entire data set, given the model parameters, is

$$\mathcal{P}(\mathcal{D}|\Lambda) = \prod_{i=1}^{N} \mathcal{P}(t_i|F_i, \Lambda). \tag{S47}$$

To find the best-fit parameter set Λ, we maximize the log-likelihood function $\mathcal{L} = \ln \mathcal{P}(\mathcal{D}|\Lambda)$,

$$\mathcal{L} = \sum_{i=1}^{N} \ln \left. \frac{d\Sigma_{F_i}(t_i)}{dt} \right|_{\Lambda}, \tag{S48}$$

where we have neglected an additive constant dependent on δt that does not affect the fitting.

To prevent the maximization algorithm, implemented in *Mathematica*, from veering into unphysical regions of parameter space, the parameters were constrained to vary over physically sensible ranges: E_0, H, G, d, $r_0 \geq 0$, $\alpha_{\max} > \alpha_c > \alpha_{\min} + \gamma$. Here the buffer angle γ was set to 5°, to put a constraint on the minimum possible angular range for the small α conformational state. This choice of γ was based on the magnitude of fluctuations in molecular dynamics trajectories of α in Ref. [6], though other choices of γ within a few degrees also lead to similar maximum log-likelihoods and best-fit parameter sets Λ.

[1] van Kampen, N. G. *Stochastic processes in physics and chemistry* (Elsevier, Amsterdam, 2007).

[2] Chakrabarti, S., Hinczewski, M. & Thirumalai, D. Plasticity of hydrogen bond networks regulates mechanochemistry of cell adhesion complexes. *Proc. Natl. Acad. Sci.* **111**, 9048–9053 (2014).

[3] Barsegov, V. & Thirumalai, D. Dynamics of unbinding of cell adhesion molecules: Transition from catch to slip bonds. *Proc. Natl. Acad. Sci.* **102**, 1835–1839 (2005).

[4] Chakrabarti, S., Hinczewski, M. & Thirumalai, D. Phenomenological and microscopic theories for catch bonds. *J. Struct. Biol.* **197**, 50–56 (2017).

[5] Buckley, C. D. *et al.* The minimal cadherin-catenin complex binds to actin filaments under force. *Science* **346**, 1254211 (2014).

[6] Li, J. *et al.* Structural determinants of the mechanical stability of α-catenin. *J. Biol. Chem.* **290**, 18890–18903 (2015).
